# Hypoglycemia aggravates cognitive degeneration by activating endothelial ZBP1-mediated PANoptosis in type 2 diabetic mice

**DOI:** 10.3389/fphar.2026.1825677

**Published:** 2026-07-01

**Authors:** Wenping Luo, Qian Xiao, Na Li

**Affiliations:** 1 Department of Geriatrics, Laboratory of Research and Translation for Geriatric Diseases, The First Affiliated Hospital of Chongqing Medical University, Chongqing, China; 2 Department of Cardiology, The First Affiliated Hospital of Chongqing Medical University, Chongqing, China

**Keywords:** AGE- RAGE axis, cognitive degeneration, diabetes, hypoglycemia, PANoptosis, ZBP1

## Abstract

**Introduction:**

Recurrent hypoglycemia increases cognitive impairment in diabetic patients. Following cerebral neuron injury, endothelial cells provide morphological, metabolic, and immune support to damaged neurons, but the inflammatory mechanism underlying hippocampal neuron degeneration remains unclear.

**Methods:**

The Morris water maze test was performed to measure cognitive changes in type 2 diabetic mice. ZBP1 expression was knocked down via small interfering RNA transfection in bEnd.3 brain endothelial cells.

**Results:**

PANoptosis, a defined form of programmed cell death (PCD), was increased by hypoglycemia in the hippocampus of diabetic mice in vivo and by low glucose in bEnd.3 cells in vitro. ZBP1 knockdown reduced low-glucose-induced PANoptosis in high-glucose-cultivated bEnd.3 cells. RNA transcriptomics sequencing revealed that AGE-RAGE signaling was significantly altered after ZBP1 knockdown, which was confirmed by biochemical data.

**Discussion:**

Hypoglycemia impairs cognition in diabetic mice by activating brain endothelial ZBP1-mediated PANoptosis via the AGE-RAGE axis. Targeting ZBP1 may represent a novel therapeutic strategy for diabetes-associated cognitive dysfunction.

## Highlights

- PANoptosis is significantly induced by recurrent hypoglycemia in diabetic mouse hippocampus.
- ZBP1, a sensor of the PANoptosome, was specifically activated by low glucose in brain endothelial cells.
- Hypoglycemia activated ZBP1-PANoptosis via AGEs-RAGE axis.
- Inhibiting ZBP1-PANoptosis and the AGE-RAGE axis may be a potential approach to prevent hypoglycemia-induced cognitive degeneration in type 2 diabetes.

## 1 Introduction

The harmful effects of hypoglycemia on the course of type 1 (Kaur and Seaquist, 2023) and type 2 diabetes (International Hypoglycaemia Study, 2019) are multifaceted, especially the irreversible damage to the brain and cognitive function (Verhulst et al., 2022). There are multiple causes of hypoglycemia, but the main causes include improper insulin injection (Galvez et al., 2023) or glucose metabolism disorders after surgery (Sandoval and Patti, 2023). Although mild or moderate hypoglycemia is not life-threatening, it can cause cognitive impairment if it occurs repeatedly (Zhou et al., 2018). The brain is extremely sensitive to fluctuations in blood glucose. Studies have indicated that recurrent hypoglycemia (RH) leads to cognitive impairment in diabetic patients (McNay and Cotero, 2010) or diabetic mice (Gao et al., 2021); however, the underlying mechanisms remain unclear.

Endothelial cells are the most important cells for maintaining the morphology and functions of blood vessels. Affected by blood glucose fluctuations (Zhang et al., 2014), endothelial cells secrete various signaling factors to participate in the processes of nerve signal transmission, neuronal activity and other types of humoral regulation (Chen et al., 2021). Activated c-Fos/focal adhesion preserved endothelial integrity and alleviated diabetic retinopathy (Ge et al., 2024). c-Fos, a marker of neuronal activation, is involved in the processes of memory formation and learning and also regulates neural plasticity (Zhang et al., 2002). The hippocampus is a major organ involved in spatial learning and memory formation (El Mahmoudi et al., 2023). Won et al. (2012) reported that moderate hypoglycemia caused hippocampal dendrite damage, microglial activation, and cognitive impairment in rats with type 1 diabetes (Won et al., 2012). Most studies have focused on type 1 diabetes during episodes of hypoglycemia; however, type 2 diabetes accounts for a greater proportion of people with clinical diabetes. To provide more reliable evidence for better standardized blood glucose management and prevention of brain damage caused by hypoglycemia, it is necessary to understand the cognitive changes and related mechanisms in type 2 diabetes mellitus (T2DM) patients or mice during hypoglycemic episodes.

Inflammation is closely related to both energy metabolism in the brain and cognitive impairment (Kealy et al., 2020). The authors reported that hypoglycemia causes widespread inflammation throughout the body and may lead to serious adverse consequences such as cognitive impairment and dementia. The underlying molecular mechanism remains to be studied. PANoptosis is a new type of CD revealed in recent years and refers to a unique inflammatory cell death modality involving interactions among pyroptosis, apoptosis, and necroptosis. PANoptosis can be mediated by multifaceted PANoptosome complexes assembled by integrating components from other cell death modalities (Sun et al., 2024). Z DNA binding protein (ZBP1) has been characterized as a critical innate immune sensor of not only viral RNA products but also endogenous nucleic acid ligands. ZBP1 sensing of the Z-RNA produced during influenza virus infection induces cell death in the form of pyroptosis, apoptosis, and necroptosis (PANoptosis) (Zheng and Kanneganti, 2020; Lee et al., 2021). The functions of these molecules in innate immunity and inflammatory cell death suggest new therapeutic targets for ZBP1-mediated diseases. A previous study revealed the progression of PANoptosis in diabetes mellitus and complications from diabetes (Han et al., 2025), including cardiovascular diseases (Chang et al., 2024) and diabetic kidney injury (Lv et al., 2025). However, whether PANoptosomes form in the context of hippocampal injury induced by hypoglycemia is unclear. In this study, the effects of hypoglycemia on mice with type 2 diabetes were assessed *in vivo* and *in vitro*, and we hope that these findings provide a promising landscape for studying diabetes treatment.

## 2 Materials and methods

### 2.1 Animals

Male 8-week-old C57BL/KsJ (C57) and BKS-*db/db* mice were acquired from the Experimental Animal Center of Chongqing Medical University. They were stored in a 12 h light–dark cycle at 22 °C–25 °C with free access to food and water. After 1 week of adaptive feeding, the C57 mice were randomly assigned into the C57 group (n = 6) and the C57-RH group (n = 6). The *db/db* mice were randomly assigned into the DM group (n = 6) and the DM-RH group (n = 6). All animal experiments in this study were approved by the Laboratory Animal Management and Utilization Committee of Chongqing Medical University (Approval number: SC14,15-DHETK Chongqing 2007–0001). And it was carried out strictly in accordance with the guidelines for the care and use of laboratory animals issued by the National Institutes of Health.

### 2.2 Animal hypoglycemia models

The mice of hypoglycemia group were subcutaneous injected with insulin (Wanbang, China) with 10.0 units/kg at 8:00–9:00 after overnight fasting (Luo et al., 2022). The Sham group was administered the same volume of PBS (hypoglycemia for 1 h/5 days). After insulin was injected, blood glucose were detected every 30 min to guarantee the maintenance of glucose levels in severe hypoglycemia (≤3.9 mmol/L) (Luo et al., 2022). Mice were injected with 20% glucose for hypoglycemia episode termination. None of them had seizures or coma during a hypoglycemia attack.

### 2.3 Morris water maze experiment

After the hypoglycemic model was established, we conducted a water maze experiment on each group of mice. According to the methods reported in the literature (Balci et al., 2025), there are three phases in the experiment. Adaptation period: Let the animals swim freely in the pool and get familiar with the water maze environment. Learning stage: Put the animal into the water maze, let it find and remember the location of the platform, record the time (incubation period) and track of the animal to find the platform. Test phase: Remove the platform, let the animals swim in the pool for a certain period of time, and record the time and trajectory of the animals near the previous platform location. All of mice were sacrificed and tissues were collected after behavioral testing (Yu et al., 2025).

### 2.4 Immunohistochemistry

Based on the literature (Chang et al., 2026), the brain sections were incubated ter dewaxing and rehydration. Antigen retrieval was performed using the EDTA method, and the sections were blocked with 5% goat serum at room temperature. The brain sections of mice were incubated with the primary antibody of CD45 (1:100, Zenbio, R380923) at 4 °C overnight. The second day, the sections were incubated with corresponding secondary antibodies at room temperature for 1 h, followed by staining using a 3,3′-diaminobenzidine (DAB) kit (Solarbio, Beijing, China). Nuclei were counterstained with hematoxylin, and the sections were then dehydrated, cleared, and mounted. Finally, images were observed and captured under a microscope, and the mean optical density (IOD/Area) of positive signals was analyzed using Image-Pro Plus software.

### 2.5 Immunofluorescent staining

The dewaxing, antigen retrieval and blocking processes of paraffin sections are the same as above. Primary antibodies (c-FOS, ZENBIO, 340249; NeuN, ZENBIO, R25129; Caspase1, Proteintech, 22915-1-AP; Caspase8, ZENBIO, 680093; RIPK3, ZENBIO,505431; RAGE, ABclonal, A26429PM) were incubated overnight at 4 °C. The second day, sections were incubated with corresponding secondary fluorescence antibodies for 1 h. Diluted DAPI was added and incubated for 10min at room temperature. After three times of TBST washing, sections were observed by fluorescence microscope. The imaging acquisition parameters are as follows: Microscope (Leica DMi8); exposure time and gain value in each independent experiment should keep consistent. Laser power was in 1%–5%.

### 2.6 Hematoxylin and eosin (H&E) staining

Mouse brain sections dewaxed in xylene and rehydration through a graded ethanol series, the sections were stained with hematoxylin for 10 min, rinsed under running water, briefly differentiated in 1% hydrochloric acid alcohol, and immediately rinsed again under running water. The sections were then placed in water for bluing until the nuclei appeared blue. Subsequently, the sections were immersed in 0.5% eosin solution for 1–3 min for cytoplasmic staining. After dehydration through a graded ethanol series and clearing in xylene, the sections were mounted with neutral balsam. Pathological changes in the lung tissues, including alveolar wall thickness, congestion and edema, inflammatory cell infiltration, and structural damage, were observed under a light microscope.

### 2.7 Cell culture and treatment

The bEnd3 cell line (TCM-C715, HyCyte, China) was cultivated at 37 °C with 5% CO_2_ in cultured medium. High-glucose medium was composed of DMEM, 35 mmol/L glucose supplemented with 10% FBS and 1% penicillin/streptomycin. Low-glucose medium was composed of DMEM, 3 mmol/L glucose supplemented with 10% FBS and 1% penicillin/streptomycin. Based on the published literature, the HG + LG group was treated with high-glucose medium for 24 h, then cultured with low-glucose medium for 3 h, and then replaced with high-glucose medium for another 6 h. This process was repeated 5 times to modulate the process of recurrent hypoglycemia (Zuo et al., 2025). The HG group was cultured by high-glucose medium consistently.

### 2.8 Small interfering RNA transfection

The Bend.3 cells were incubated in the six-well plate 1 day before transfection. The second day, cells were transfected with 50 nM siRNA or negative control according to the manufacturer’s guidelines (RM09014P, ABclonal, China). Cells were added to each well with 8 μL transfection reagent. After 6 h, cultured medium was half-replaced with fresh completed medium. After 48 h, total protein was extracted using Western blot analysis. The siRNA sequences for AIM2 were 5′-GCAGUGACAAUGACUUUAATT-3′and 3′-UUA​AAG​UCA​UUG​UCA​CUG​CTT-5′. The siRNA sequences for RIPK1 were 5′-GGC​AGA​AUG​AGG​CUU​ACA​ATT-3′ and 3′-UUG​UAA​GCC​UCA​UUC​UGC​CTT-5′. The siRNA sequences for ZBP1 were 5′-GAGACAAUCUGGAGCAAAATT3′and 3′-UUU​UGC​UCC​AGA​UUG​UCU​CTT-5′, respectively. The siRNA sequences for RAGE were 5′-ACC​GGG​GGC​ATT​CAG​CT-3′ and 3′-TCT​AAA​AAA​GGG​CAT​TCA​GCT​GT-5′. The negative control sequences for those small interfering RNA were sense strand: 5′-UUC​UCC​GAA​CGU​GUC​ACG​UTT-3′ and antisense strand: 5′-ACG​UGA​CAC​GUU​CGG​AGA​ATT-3'.

### 2.9 RNA sequencing

Total RNA was extracted from bEnd.3 transfected with siNC or siZBP1 after HG + LG treatment using the TRIzol reagent (Invitrogen) according to the manufacturer’s instructions. A total amount of 1 μg RNA was used to prepare the sequencing libraries using an NEBNext Ultra RNA Library Prep Kit for Illumina (NEB, Ipswich, MA) following the manufacturer’s recommendations. The qualified libraries were sequenced on an Illumina NovaSeq 6000 platform to generate 150 bp paired-end reads by a commercial service provider, like Novogene/Gene *Denovo*. Differential gene expression analysis between siNC and siZBP1 groups was performed using the DESeq2 or edgeR package in R, with genes showing a |log2(Fold Change) |> X and an adjusted p-value (FDR) < 0.05 considered statistically significant. Gene Ontology (GO) enrichment and Kyoto Encyclopedia of Genes and Genomes (KEGG) pathway analysis of the differentially expressed genes (DEGs) were conducted using the clusterProfiler package in R, with an adjusted p-value <0.05 considered significant.

### 2.10 Flow cytometry

The gathered cells were rinsed with PBS and 2% fetal calf serum. Each examination was performed in non-dyed, annexin V, PI, and PI and annexin V double-dyed groups. The treatment group underwent double dyeing from low to high. The 4× binding buffer was diluted to 1×buffer with PBS, and after the residual PBS in the centrifuge tube was absorbed, 100 μL of 1×binding buffer was added to each tube, and the cells were blown with a pipette to fully resuspend the cells, whereas the dyes were added under the condition of dark light. Annexin V or PI 5 μL was added to the single-dyed and double-dyed groups and gently mixed with a pipette. After the incubation at room temperature and darkness for 15 min and mixing with 1×binding buffer 300 μL, the cell suspension was removed to a 5 mL flow tube under dark light and was analyzed by flow cytometry within 1 h. Annexin V-FITC/PI apoptosis detection kit was used as a probe with annexin V annexin V-labeled FITC. The maximum excitation wavelength of FITC was 488 nm, the maximum emission wavelength was 525 nm, and the green fluorescence of FITC was found in the FL1.

### 2.11 Western blotting

Total protein was extracted from mouse hippocampus tissues or Bend.3 cells, and protein concentration was detected using the BCA method as manufacturer’s guidance (Beyotime, P0011). Equal amounts of protein samples were separated by SDS-PAGE and transferred to PVDF membranes. After blocking with 5% skim milk for 1h, the membranes were incubated with specific primary antibodies (NLRP3, ZENBIO, R381207; RIPK1, ZENBIO, R25593; P-RIPK1, ZENBIO, R10014; RIPK3, ZENBIO, 620375; P-RIPK3, ZENBIO, R30283; Casepase-8, Proteintech, 13423-1-AP; Caspase-1, Proteintech, 22915-1-AP; ZBP1, ZENBIO, 856001; AIM2, Proteintech, 20590-1-AP; N-GSDMD, Proteintech, ab215203; GSDMD, ZENBIO, R40136; MLKL, ZENBIO, R380559; Caspase-3, Proteintech, 66470-2-Ig; FADD, ZENBIO, R24274; ASC, ZENBIO, 340097; IL-18, ZENBIO, 516737; IL-1β, Proteintech, ab283818; Casepse-9, Proteintech, 66169-1-Ig; Bcl2, ZENBIO, 381702; BAX, ZENBIO, R380709; β-actin, Servicebio, GB15001-100; RAGE, ZENBIO, R25540) at 4 °C overnight. The second day, the membranes were washed with TBST and were incubated with corresponding HRP-conjugated secondary antibodies at room temperature for 1 h. Protein bands were visualized using an ECL chemiluminescence detection reagent in a chemiluminescence imaging system. Each molecule needs to be uniformly normalized based on the expression level of the internal reference before comparison. The gray value was quantified using ImageJ software.

### 2.12 Cell viability assay

The cell viability was detected by Cell Counting Kit-8 (CCK8, HY-K0301, Med Chem Express, USA) assay. The Bend3 cells were seeded in 96-well growth medium plate overnight at 1*10^4^ cells/well. After 24 h, cells were administrated by different conditions and were maintained at 37 °C and 5% CO_2_ in a humidified incubator, then cells were cultured with CCK-8 for 30 min. Ultimately, the cell activity was calculated by reading the OD value at a wavelength of 450 nm in each well.

### 2.13 Statistical analysis

All data were shown as mean ± SEM. Statistical analyses were performed using GraphPad Prism software (version 9.0). Comparisons between two groups were conducted using Student’s t-test. For comparisons among multiple groups, one-way analysis of variance (One-way ANOVA) was employed, followed by Tukey’s *post hoc* test. A *P* < 0.05 was regarded as statistically significant. Data from at least three independent experiments were evaluated (Tataei et al., 2023).

## 3 Results

### 3.1 Recurrent hypoglycemia exacerbates cognitive dysfunction of the diabetic mice

**FIGURE 1:**
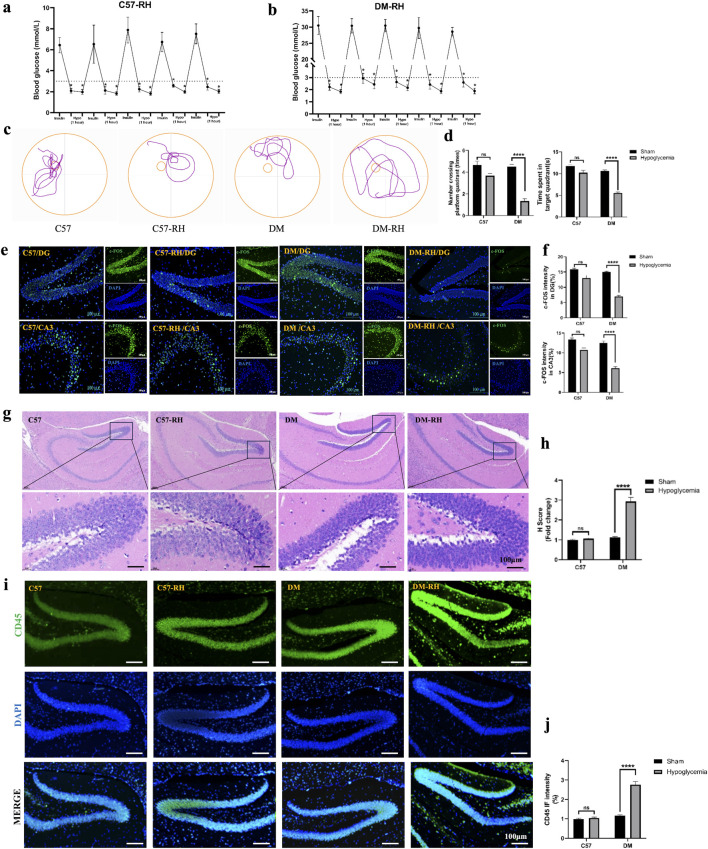
Recurrent hypoglycemia aggravates cognitive degeneration in type2 diabetic mice. **(a,b)** Blood glucose record of the C57-RH group (n = 6) and the DM-RH group (n = 6). **(c)** Representative images of Morris water maze traces of mice in the C57, C57-RH, DM, DM-RH groups. **(d)** Statistical analysis of number crossing the platform quadrant and the time spent in target quadrant in the Morris water maze experiment (n = 6). **(e,f)** Immunofluorescent staining and statistical analysis of c-FOS (a marker of neuronal activity) of mouse hippocampus in different groups (n = 6). **(g,h)** H&E staining and histological score (H score) of mouse hippocampus in different groups. **(i,j)** Immunofluorescent staining and statistical analysis of CD45 of mouse hippocampus in different groups (n = 6). ns, no significance; ****p<0.0001 vs. the DM group.

We established a model of recurrent hypoglycemia in C57 and type 2 diabetes mice, respectively. The blood glucose of C57-RH was shown in Figure 1a, while the blood glucose of the DM-RH group was shown in Figure 1b. The blood glucose concentration of the DM group was consistently higher than 20 mmol/L, much higher than the criteria for a diabetes diagnosis (random blood glucose concentration ≥11.1 mmol/L). However, after subcutaneous injection of insulin, the blood glucose concentration of the mice in the C57-RH and the DM-RH group was reduced to less than 3.9 mmol/L and maintained for 1 h. These results indicated that the hypoglycemic model has been successfully established.

The effects of hypoglycemia on spatial learning and cognitive function were evaluated by the Morris water maze experiment (Lissner et al., 2021). Representative images of the mice swimming trajectory in different groups are shown in Figure 1c. The results suggested that there was no significantly deference between the C57 and the C57-RH group. However, compared with the DM group, the number of crossing platform quadrant sharply reduced in the DM-RH group (*n* = 6; *p*<0.0001). In addition, the time spent in platform quadrant reduced significantly in the DM-RH group related to the DM group (*n* = 6; *p*<0.0001) (Figure 1d). These results indicated that hypoglycemia impaired the cognitive ability of the DM mice rather than the C57 mice. The hippocampus plays a critical role in cognition (Lisman et al., 2017). We conducted immunofluorescent staining of c-FOS in hippocampus (Figure 1e), which is an important marker of neuronal activity and memory formation (Zhang et al., 2002). The results showed that compared with the DM group, the density of c-FOS in the DG area and the CA3 area of the hippocampus in the DM-RH group sharply decreased, respectively (*n* = 6; *p*<0.0001) (Figure 1f). Additionally, H&E staining of hippocampus showed a higher histological score (H score) in the DM-RH mice than the DM group, however, there was no significantly difference between the C57 group and the C57-RH group (Figures 1g,h). Moreover, we conducted the IHC staining of CD45 antibody in the mice hippocampus (Figure 1i). The results showed that the infiltration of lymphocytes in the hippocampus increased in the DM-RH group compared with the DM group, which suggests hypoglycemia induced inflammation in the hippocampus of diabetic mice. Subsequently, we specifically explored the mechanisms of impaired cognition in diabetic mice induced by hypoglycemia.

### 3.2 Recurrent hypoglycemia aggravates PANoptosis in hippocampus of the diabetic mice *in vivo* and in brain endothelial cells *in vitro*

**FIGURE 2:**
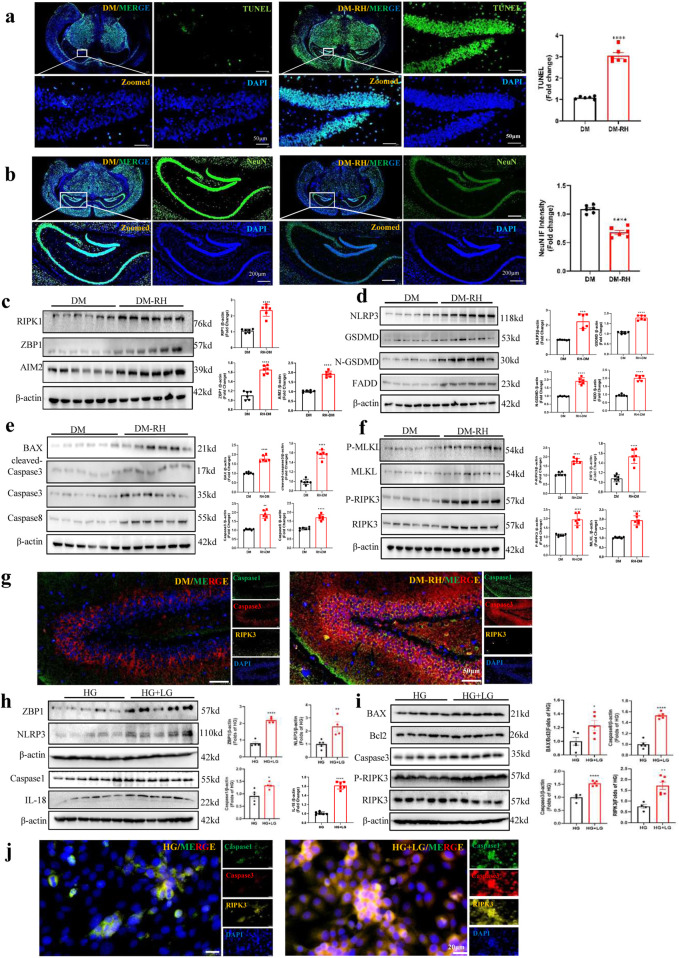
Hypoglycemia exacerbates PANoptosis in type 2 diabetic hippocampus *in vivo* and in bEnd.3 *in vitro*. **(a)** TUNEL staining of mouse hippocampus in the DM and the DM-RH groups. Scale bar = 50 μm. **(b)** Immunofluorescent staining of NeuN in mouse hippocampus in the DM and the DM-RH group (n = 6). Scale bar = 100 μm.** (c)** Western blotting and statistical analysis of PANoptosme receptors in hippocampus of type 2 diabetic mice (*n* = 6). **(d)** Western blotting and statistical analysis of pyroptosis-associated markers in hippocampus of type 2 diabetic mice (*n* = 6). **(e)** Western blotting and statistical analysis of apoptosis-associated markers in hippocampus of type 2 diabetic mice (*n* = 6). **(f)** Western blotting and statistical of necroptosis-associated markers in hippocampus of type 2 diabetic mice (*n* = 6). **p<0.01 vs. DM group; ***p<0.001 vs. DM group; ****p<0.0001 vs. the DM group. **(g)** Immunofluorescent staining of Caspase1, Caspase3 and RIPK3 in the hippocampus of the DM and the DM-RH mouse. **(h,i)** Western blotting and statistical analysis of pyroptosis, apoptosis and necroptosis markers in bEnd.3 treated by HG or HG + LG (*n* = 6). **(j)** Immunofluorescent co-localization staining of Caspase1, Caspase3 and RIPK3 in the bEnd.3 after HG or HG + LG treatment. *p<0.05, **p<0.01, ****p<0.0001 vs. the HG group.

The mechanism of cognitive dysfunction induced by hypoglycemia in diabetic mice remains unclear. Previous studies suggest that hypoglycemia aggravates inflammation in the hippocampus (McNeilly et al., 2016). Therefore, we tested whether programmed cell death (PCD) was induced by hypoglycemia in the mice hippocampus. As shown in Figure 2a, TUNEL staining indicated that hypoglycemia exacerbated apoptosis in hippocampus of the diabetic mice. In addition, IF staining of NeuN showed that hypoglycemia significantly exacerbated neuron impairment in diabetic mouse (Figure 2b, n = 6; *p*<0.0001). Furthermore, receptors of PANoptosomes, including Receptor-interacting protein kinase 1 (RIPK1), Z-DNA binding protein 1 (ZBP1) and Absent in Melanoma 2 (AMI2), were activated in DM-RH group compared with the DM group, respectively (Figure 2c, n = 6; *p*<0.0001). The expression of the pyroptosis markers, including NLRP3, N-terminal of Gasdermin D (GSDMD), and Fas-associating via death domain (FADD) was increased in the hippocampus of the DM-RH group compared with the DM group (Figure 2d, *n* = 6; *p*<0.0001). The expression of apoptosis markers, including BAX, cleaved-Caspase3, Caspase-3 and Caspase-8, was elevated in the DM-RH group than the DM group (Figure 2e, *n* = 6; *p*<0.0001). Moreover, the expression of necroptosis markers, including phosphorylated and total Receptor-interacting protein kinase 3 (RIPK3), Mixed lineage kinase domain-like protein (MLKL), obviously increased in the DM-RH group compared with the DM group (Figure 2f, *n* = 6; *p*<0.0001). Representative IF images of Caspase1, Caspase3 and RIPK3 in the mouse hippocampus are shown in Figure 2g. The intensity and the colocalization of Caspase1, Caspase3 and RIPK3 in in the DM-RH were increased in the DM-RH group compared with the DM group. Briefly, PANoptosis including pyroptosis, apoptosis and necroptosis, was significantly activated by hypoglycemia in hippocampus of diabetic mice.

Brain endothelial cells are sensitive to blood glucose fluctuations (Clyne, 2021). We detected the effect of low glucose on brain-derived endothelial cells.3 (bEnd.3). Similarly, the expression level of PANoptosis was shown in Figures 2h,i. The expression level of ZBP1 was about 2-fold in the HG + LG group greater than that in the HG group (Figure 2h, *n* = 5; *p*<0.0001). The expression levels of NLRP3, Caspase1 and IL-18 significantly upregulated after low glucose treatment (Figure 2h, *n* = 5; *p*<0.05). The expression level of apoptosis (BAX/Bcl2 and caspase3) and necroptosis(P-RIPK3/RIPK3) was upregulated in the HG + LG group compared with the HG group, respectively (Figure 2i, *n* = 5; *p*<0.05). Compared with the HG group, the IF intensity of Caspase1, Caspase3 and RIPK3 was significantly increased in the HG + LG group (Figure 2j). In summary, PANoptosis was activated by low glucose treatment in endothelial cells *in vitro* and in mouse hippocampus *in vivo*.

### 3.3 ZBP1 knockdown alleviates PANoptosis induced by low glucose in bEnd.3 cells *in vitro*

**FIGURE 3:**
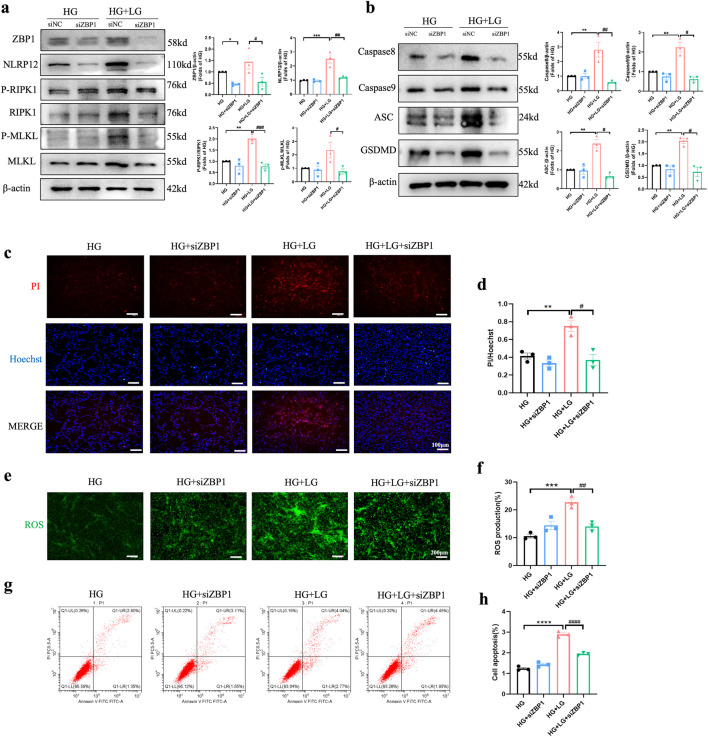
ZBP1 knockdown alleviates low glucose-induced PANoptosis and improved cell viability in bEnd.3. **(a,b)** Western blotting and statistical analysis of the pyroptosis, apoptosis and necroptosis-associated markers after ZBP1 knockdown in the HG or the HG + LG treated bEnd.3 (n = 3). **(c,d)** Hoechst/PI staining and analyzed PI/Hoechst ratio after ZBP1 knockdown in Bend.3 after HG or HG + LG treatment (n = 3). Scale bar = 100 μm **(e,f)** Representative images and statistical analysis of ROS production in bEnd.3 in different groups (n = 3). Scale bar = 200 μm. **(g,h)** Flow cytometry of Cell apoptosis in bEnd.3 after knockdown ZBP1 treated with HG or HG + LG. *p<0.05, **p<0.01, ****p<0.0001 vs. the HG group; ^#^ *p* < 0.05, ^##^ *p* < 0.001, ^###^ *p* < 0.001 vs. the HG + LG group.

To determine which sensor of PANoptosome was activated in endothelial PANoptosis, we investigated the effects of knocking down ZBP1, AIM2 and RIPK1 respectively on hypoglycemic-induced Bend3 pan-apoptosis. ZBP1 was successfully knockdown by transfected with siRNA in bEnd.3 (Supplementary Figure1a). In Figure 3a, compared with the HG group, the expression level of necroptosis (p-RIPK1, RIPK1, p-MLKL and MLKL) was increased in the HG + LG group. However, ZBP1 knockdown reversed the expression of necroptosis (*n* = 3; *p*<0.05). Similarly, ZBP1 depression sharply decreased the expression level of apoptotic markers (*n* = 3; *p*<0.01) and pyroptotic markers (Figure 3b, *n* = 3; *p*<0.05), respectively. These data collectively indicated that knocking down ZBP1 inhibited low glucose-induced PANoptosis in Bend.3. Cell apoptosis was detected by Hoechst/PI staining (Figure 3c). Statistical analysis revealed that ZBP1 knockdown attenuated apoptosis induced by low glucose in bEnd.3 cells (Figure 3d, *n* = 3; *p*<0.05). Moreover, ROS production was aggravated by low-glucose treatment in bEnd.3 cells, however, this phenomenon was reversed after ZBP1 knockdown (Figures 3e,f). The rate of cell apoptosis was shown in Figures 3g,h. Cell apoptosis was attenuated by knocking down ZBP1 after low-glucose treatment. These results collectively illustrated that ZBP1 mediated the PANoptosis and cell damage induced by hypoglycemia in brain endothelial cells.

**FIGURE 4:**
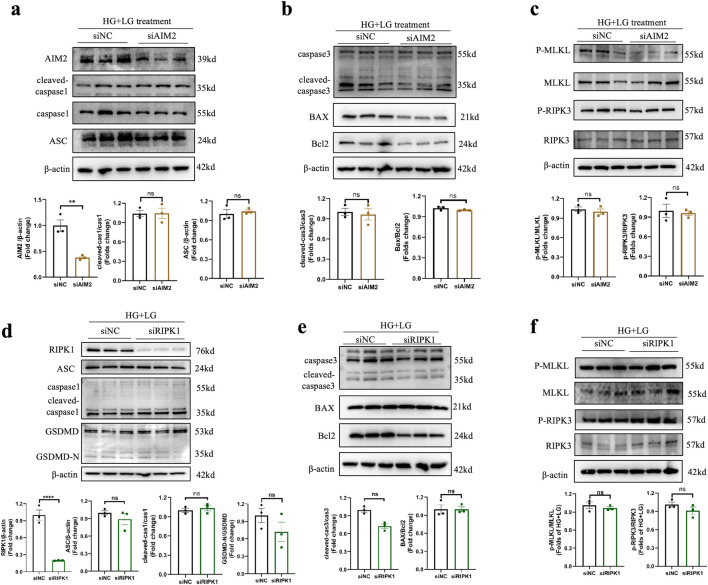
AIM2 or RIPK1 knockdown has no significant changes on the expression of PANoptosis in bEnd.3 after HG + LG pretreatment. **(a-c)** Western blotting and statistical analysis of pyroptosis, apoptosis and necroptosis in HG + LG treated bEnd.3 after AIM2 knockdown (*n* = 3). **(d-f)** Western blotting and statistical analysis of pyroptosis, apoptosis and necroptosis in HG + LG treated bEnd.3 after RIPK1 knockdown (*n* = 3). ns, no significance.

Interestingly, after knocking down the expression of AIM2 (Supplementary Figure1b; *n* = 3; *p*<0.01) or RIPK1 (Supplementary Figure1c; *n* = 3; *p*<0.001), there was no significant impact on the expression of PANoptosis-associated markers (Figures 4a–f). In summary, ZBP1 is the essential sensor of PANoptosome activated by low glucose in brain endothelial cells. Next, we explored the underlying mechanism of ZBP1-mediated hypoglycemia-induced brain endothelial PANoptosis.

### 3.4 Low glucose activated ZBP1-mediated PANoptosis via the AGEs/RAGE axis *in vitro*

**FIGURE 5:**
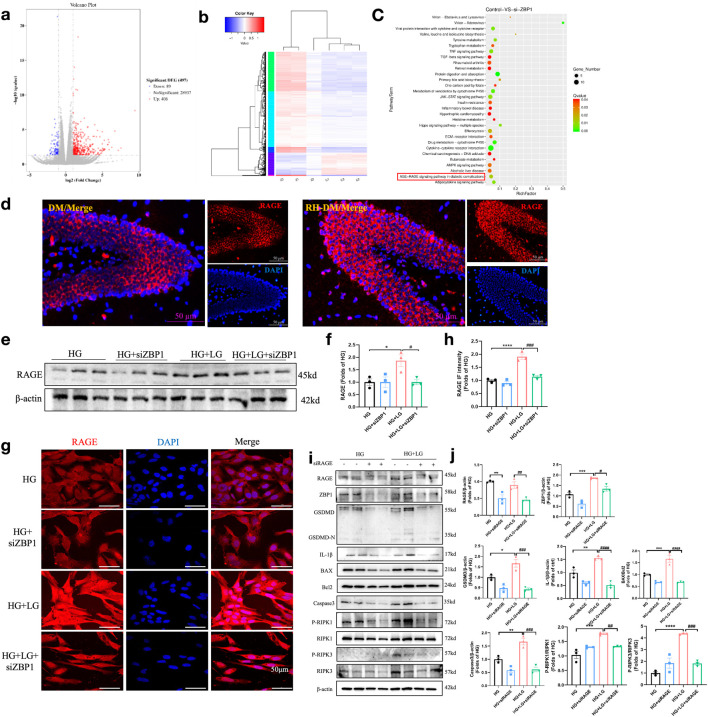
Hypoglycemia activates ZBP1-mediated PANoptosis via AGEs-RAGE axis in bEnd.3. **(a)** Volcano plot of differential expression genes (DEGs) in bEnd.3 after ZBP1 knockdown. **(b)** Heat map of DEGs in bEnd.3 after knocking down the expression of ZBP1. **(c)** KEGG enrichment analysis (bubble chart) of DEGs in bEnd.3 after ZBP1 knockdown. **(d)** Immunofluorescent staining of RAGE in mouse hippocampus of the DM and the DM-RH groups. **(e,f)** Western blotting and statistical analysis of RAGE after ZBP1 knockdown in bEnd.3 treated by HG or HG + LG (*n* = 3). **(g,h)** Representative immunofluorescent staining and statistical analysis of RAGE in bEnd.3 after ZBP1 knockdown in HG or HG + LG group (*n* = 3). **(i,j)** Western blotting and statistical analysis of RAGE and ZBP1-mediated PANoptosome after RAGE knockdown in HG or HG + LG treated bEnd.3 (*n* = 3). *p<0.05, **p<0.01, ***p<0.001, ****p<0.0001 vs. the HG group; ^#^ *p* < 0.05, ^##^ *p* < 0.01, ^###^ *p* < 0.001, ^####^ *p* < 0.0001 vs. the HG + LG group.

**FIGURE 6:**
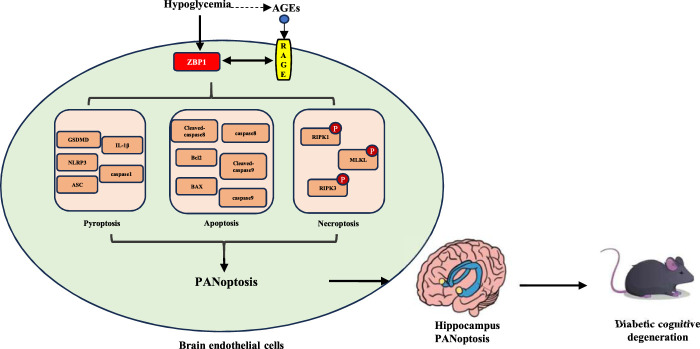
The graphical abstract of the mechanisms that hypoglycemia aggravates cognitive degeneration by activating the ZBP1-mediated PANoptosis in type 2 diabetes. In type 2 diabetic mouse brain, when hypoglycemia happens, ZBP1 is initially activated in the endothelial cells. Then hypoglycemia aggravates ZBP1-mediated PANoptosis in endothelium and hippocampus. Moreover, ZBP1 upregulates the expression of the AGEs-RAGE axis, which mediates PANoptosis in endothelial cells. Finally, endothelial cells and hippocampus were injured, which led to cognitive degeneration.

RNA transcriptome sequencing was conducted in bEnd.3 cells after ZBP1 knockdown coupled with HG + LG treatment. The volcano plot and heatmap of differentially expressed genes (DEGs) were shown in Figures 5a,b, respectively. Among DEGs, 28937 genes were upregulated, and 89 genes were downregulated. KEGG enrichment analysis revealed that the activity of the AGE-RAGE signaling pathway, which is closely related to diabetic complications, significantly changed (Figure 5c). Immunofluorescence staining showed that the intensity of RAGE in hippocampus was incrased in the DM-RH group related to the DM group (Figure 5d). In addition, Western blotting indicated that ZBP1 depression inhibited the expression of RAGE induced by HG + LG *in vitro* (Figures 5e,f) (*n* = 3; *p*<0.05). Moreover, immunofluorescence showed that the intensity of RAGE was upregulated by low glucose in bEnd.3 compared with the HG group (Figure 5g). However, ZBP1 knockdown reversed the IF intensity of RAGE (Figure 5h) (*n* = 3; *p*<0.001). Next, we detected the role of RAGE in PANoptosis induced by low glucose *in vitro*. RAGE was successfully knockdown by siRNA transfection (Supplementary Figure1d). The expression level of ZBP1-mediated PANoptosis was reversed after RAGE knockdown in HG + LG-treated Bend.3 (Figures 5i,j; *n* = 3, *p*<0.05). These results collectively indicated that hypoglycemia exacerbated ZBP1-mediated endothelial PANoptosis via activating the AGE-RAGE axis, which in turn aggravates the PANoptosis (Figure 6).

## 4 Discussion

Our study reveals the role of ZBP1-mediated PANoptosis in cognitive degeneration induced by recurrent hypoglycemia in type 2 diabetic mice. In the Graphical Abstract (Figure 6), we described the mechanism of cognitive depression induced by hypoglycemia in mice with type 2 diabetes. To be specific, ZBP1 is initiated by hypoglycemia and mediates endothelial PANoptosis (including pyroptosis, apoptosis and necroptosis) via the AGE-RAGE axis in hippocampus.

Previous studies have reported that hypoglycemia leads to cognitive impairment in patients with diabetes (Lin et al., 2021; Sheen and Sheu, 2016). However, another prospective study reported that moderate hypoglycemia did not increase the risk of cognitive impairment in middle-aged patients with abnormal blood glucose levels (Cukierman-Yaffe et al., 2019). The brain is very sensitive to blood glucose fluctuations, and severe hypoglycemia causes irreversible damage to the mouse brain, resulting in slow responses, reduced learning and memory ability and chronic cognitive impairment. Therefore, it is important to study the compensatory mechanism of the brain after hypoglycemia. A previous study revealed that the activation of mitochondrial autophagy alleviated cognitive degeneration caused by recurrent moderate hypoglycemia in diabetic mice (Wu et al., 2024). A recent study reported that lactic acid supplementation after hypoglycemia attenuated the cognitive dysfunction caused by recurrent moderate hypoglycemia in diabetic mice (Wu et al., 2025). There have also been reports of the use of medications to prevent cognitive impairment caused by hypoglycemia (Jackson et al., 2018). Our study supports a strong association between hypoglycemia with cognitive impairment in diabetes. The connection between acute central nervous system inflammation and lymphocyte infiltration has been reported in previous literature (Maheshwari et al., 2023). On the one hand, lymphocyte infiltration mainly indicated the disruption of the blood-brain barrier (Ewing-Crystal et al., 2025). On the other hand, it is related to programmed cell death (PCD) (Ifejeokwu et al., 2025) and cognitive impairment (Terrabuio et al., 2025). Notably, this study focused on mice with type 2 diabetes, where episodes of hypoglycemia are more subtle than those in mice with type 1 diabetes (van Mark et al., 2019). Nevertheless, cognitive impairment is induced by hypoglycemia, so it is necessary and novel to study models of type 2 diabetes as well.

Endothelial cells are closely related to cognitive impairment (Fang et al., 2023). Lu et al. reported that endothelial TFEB signaling-mediated autophagy disorders lead to microglial activation and cognitive dysfunction (Lu et al., 2023). Moreover, markers of vascular cognitive impairment and dementia are summarized in a review (Hosoki et al., 2023). Acute inflammation caused by infection and other factors can damage the endothelium and the blood‒brain barrier (Greene et al., 2024); noninfectious factors such as hypertension (Uiterwijk et al., 2016), diabetes (Phoenix et al., 2022) and other chronic injuries can cause endothelial inflammation and cognitive degradation as well. However, the specific mechanisms by which these factors lead to endothelial inflammation differ. This study further revealed the role of inflammatory PANoptosis in the inflammatory injury of endothelial cells induced by hypoglycemia, providing new insights for future research and targeted therapy.

To date, widespread apoptosis related to diabetes is involved mainly in complications such as diabetic liver injury (Neira et al., 2024) and diabetic kidney injury (Han et al., 2025). Moreover, the liver can be targeted to improve systemic glucose homeostasis in T2DM rats (Song et al., 2025). *Song* and colleagues reported that Roux-en-Y gastric bypass surgery regulates the expression of TFF3 in the liver of ZDF rats, thereby activating the PI3K/Akt pathway and improving T2DM. However, little is known about the mechanisms of PANoptosis related to the cognitive impairment caused by hypoglycemia, a complication of diabetes treatment. In this study, we further revealed that the activation of PANoptosome in brain endothelial cells is mediated by the ZBP1 sensor, suggesting that ZBP1 or blocking intracerebral inflammation may be potential targets for treatment. In contrast, a previous study revealed that following sciatic nerve injury in a streptozotocin rodent model of type I diabetes, the mobilization of RNAs into injured axons was attenuated and correlated with decreased axonal regeneration. This failure of axonal RNA localization resulted from decreased levels of the RNA binding protein ZBP1 (Jones et al., 2021). The different actions of ZBP1 may be related to the use of different animal models, the different target organs and the different degrees of inflammatory damage. However, the role of ZBP1 in hypoglycemia-related diseases has not been reported, and the specific mechanism remains unknown.

AGEs-RAGE have long been known to be involved in the pathology of pulmonary diseases (Sharma et al., 2021). Recent studies have shown the pivotal role of the AGE-RAGE axis in brain aging (Vitorakis and Piperi, 2024). The authors reported that upon binding to the signal transduction receptor RAGE, AGEs can initiate proinflammatory pathways and exacerbate oxidative stress and neuroinflammation, thus impairing neuronal function and cognition. AGE-RAGE signaling induces programmed cell death, disrupts the blood–brain barrier and promotes protein aggregation, further compromising brain health. This finding is in line with our conclusion, which revealed a proinflammatory effect of ZBP1-mediated PANoptosis via AGE-RAGE axis in hippocampus. Reduction of AGE deposition in brain tissue through novel pharmacological therapeutics shows great promise for mitigating the cognitive decline in brain aging. Recently, a study revealed an association between AGE-RAGE signaling and apoptosis in cerebral ischemia‒reperfusion (Wang et al., 2025), which suggests that inhibiting apoptosis by regulating the AGE-RAGE axis is an effective way to attenuate cerebral injury. Moreover, studies have suggested the role of RAGE inhibitors in neurodegenerative diseases (Reddy et al., 2023). Our study provides a novel direction for exploring neural degenerative diseases.

The limitations of this study are as follows. Firstly, the mechanisms of the interaction between ZBP1 and RAGE still awaits deep exploration. Secondly, this study explores the cognitive impairment effect of hypoglycemia in type 2 diabetic mice, which partially limits its application and translation in type 1 diabetic models. Thirdly, differences between animal models of hypoglycemia-related diabetes and human patients should be noted. Hypoglycemia occurs randomly and inconsistently in diabetic patients (Umpierrez and Korytkowski, 2016). However, animal models of hypoglycemic diabetic mice differ across studies (Zuo et al., 2025; Guo et al., 2025). Our study was based on a previous literature (Zuo et al., 2025). In conclusion, our study provides a new target for clinical prevention and treatment in cognitive impairment caused by hypoglycemia in diabetic patients.

## Data Availability

The original contributions presented in the study are publicly available. This data can be found here: China National Center for Bioinformation, accession number PRJCA046743.

## Supplementary material

SUPPLEMENTARY FIGURE 1 Validation of the successful knockdown the expression of ZBP1 **(a)**, AIM2** (b)**, RIPK1 **(c)** and RAGE **(d)** in bEnd.3.
