# Supplementary material for: Hypoglycemia aggravates cognitive degeneration by activating endothelial ZBP1-mediated PANoptosis in type 2 diabetic mice
Source: Front Pharmacol. 2026 Jul 1;17:1825677. doi: 10.3389/fphar.2026.1825677 (PMC13368779; doi:10.3389/fphar.2026.1825677)

Figure2c

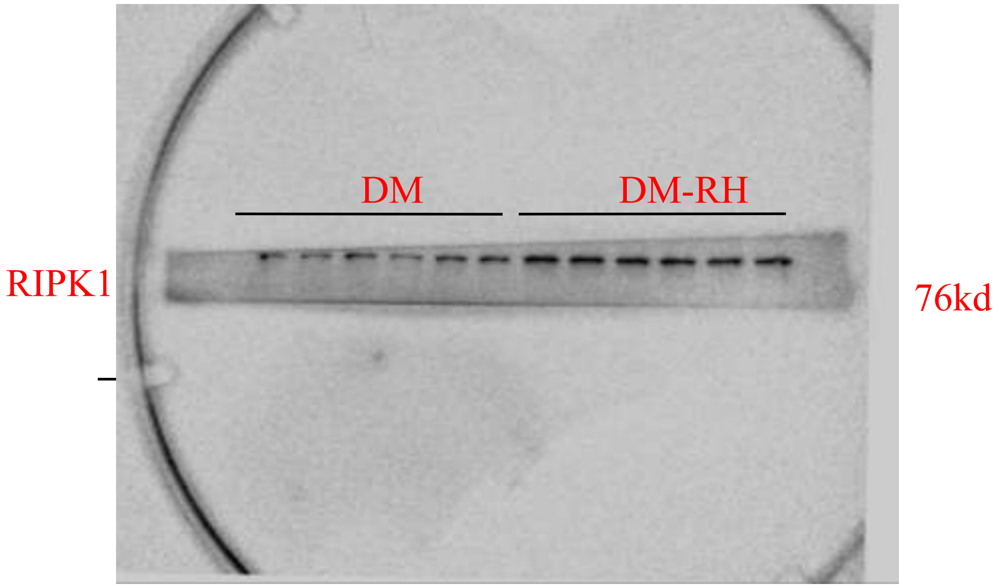

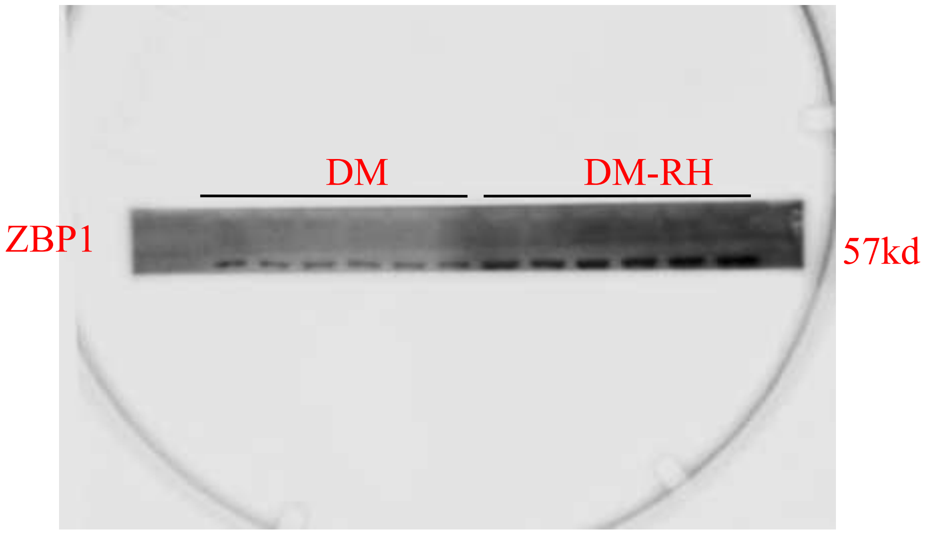

Figure2c

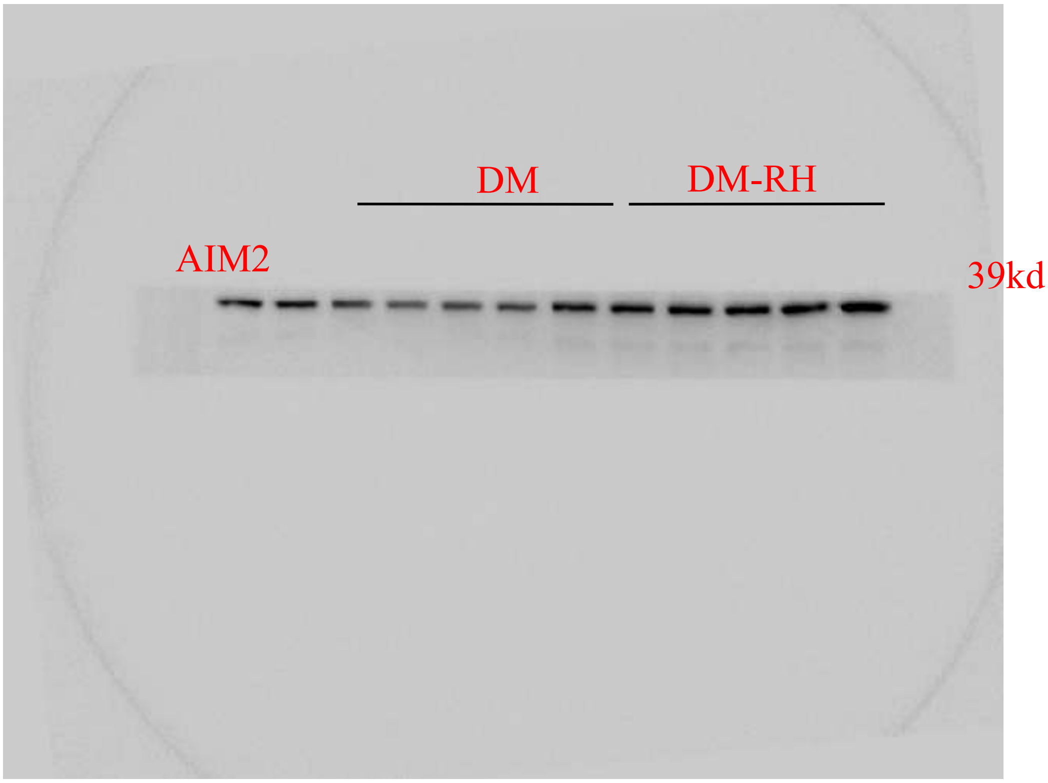

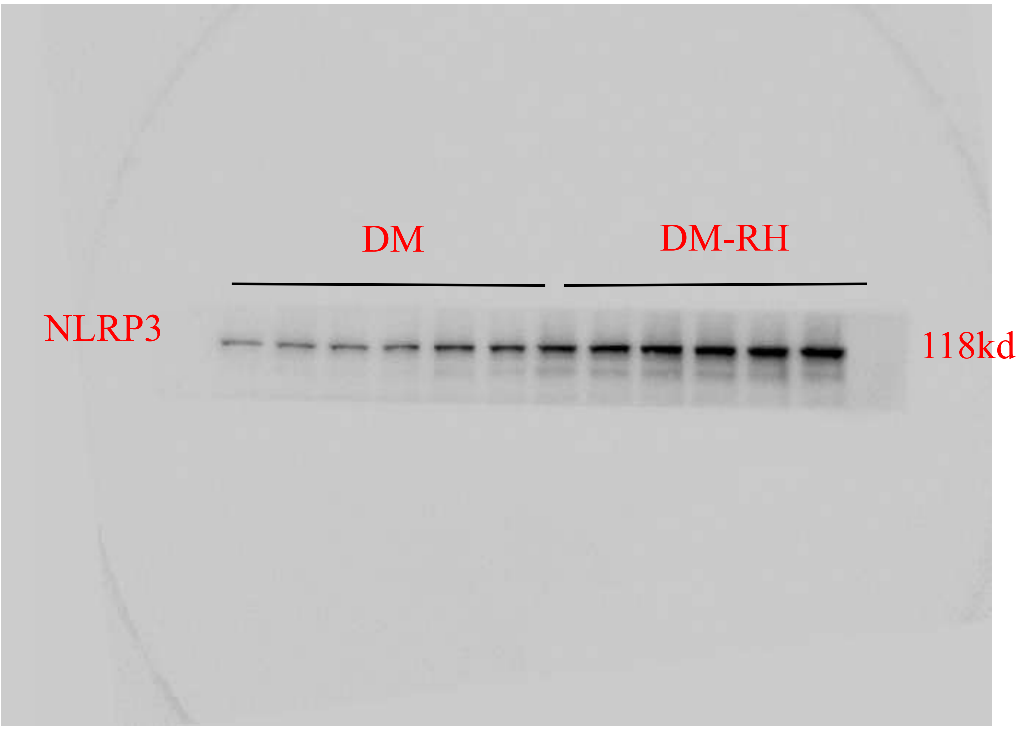

Figure2d

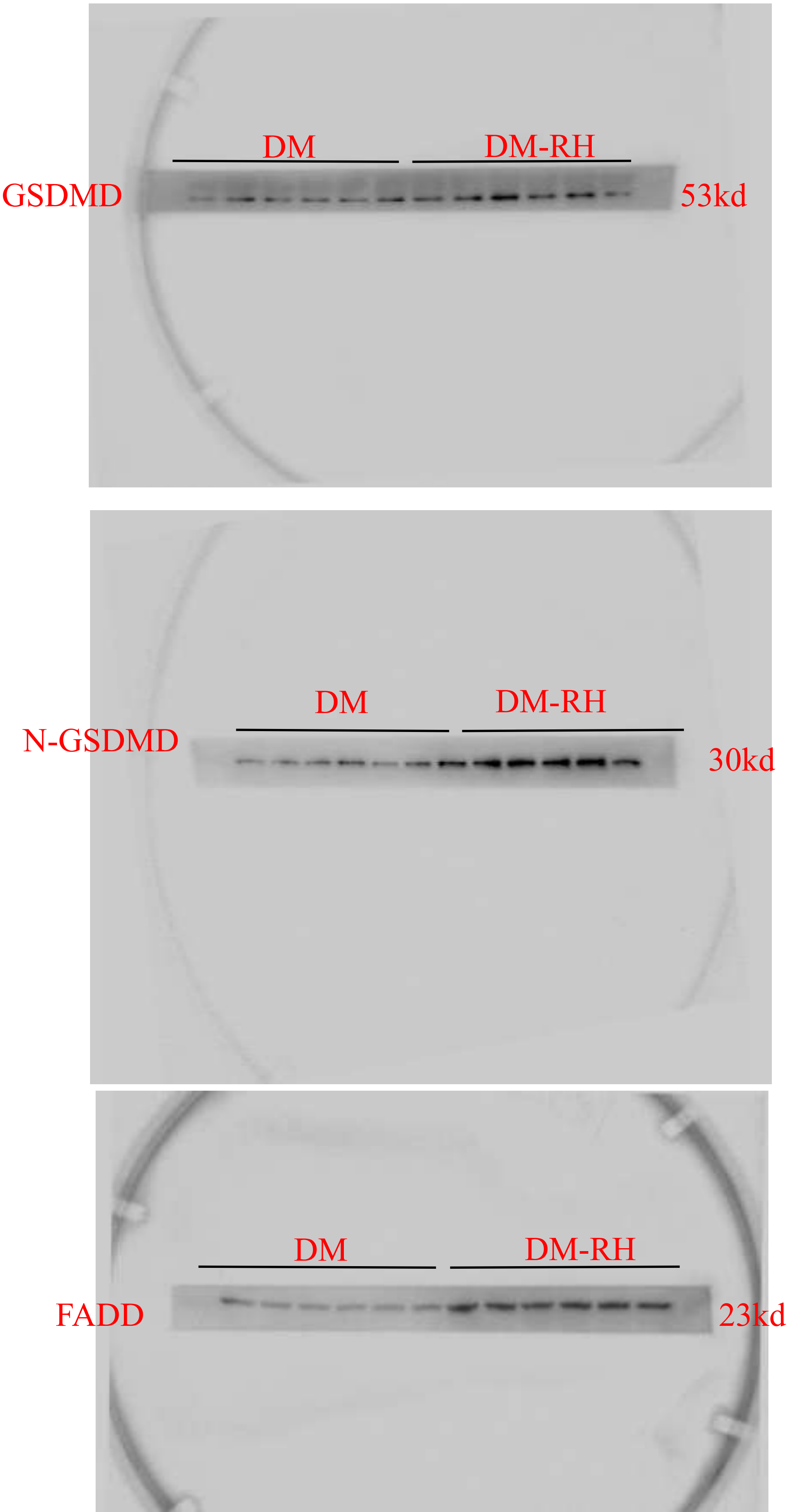

Figure2e

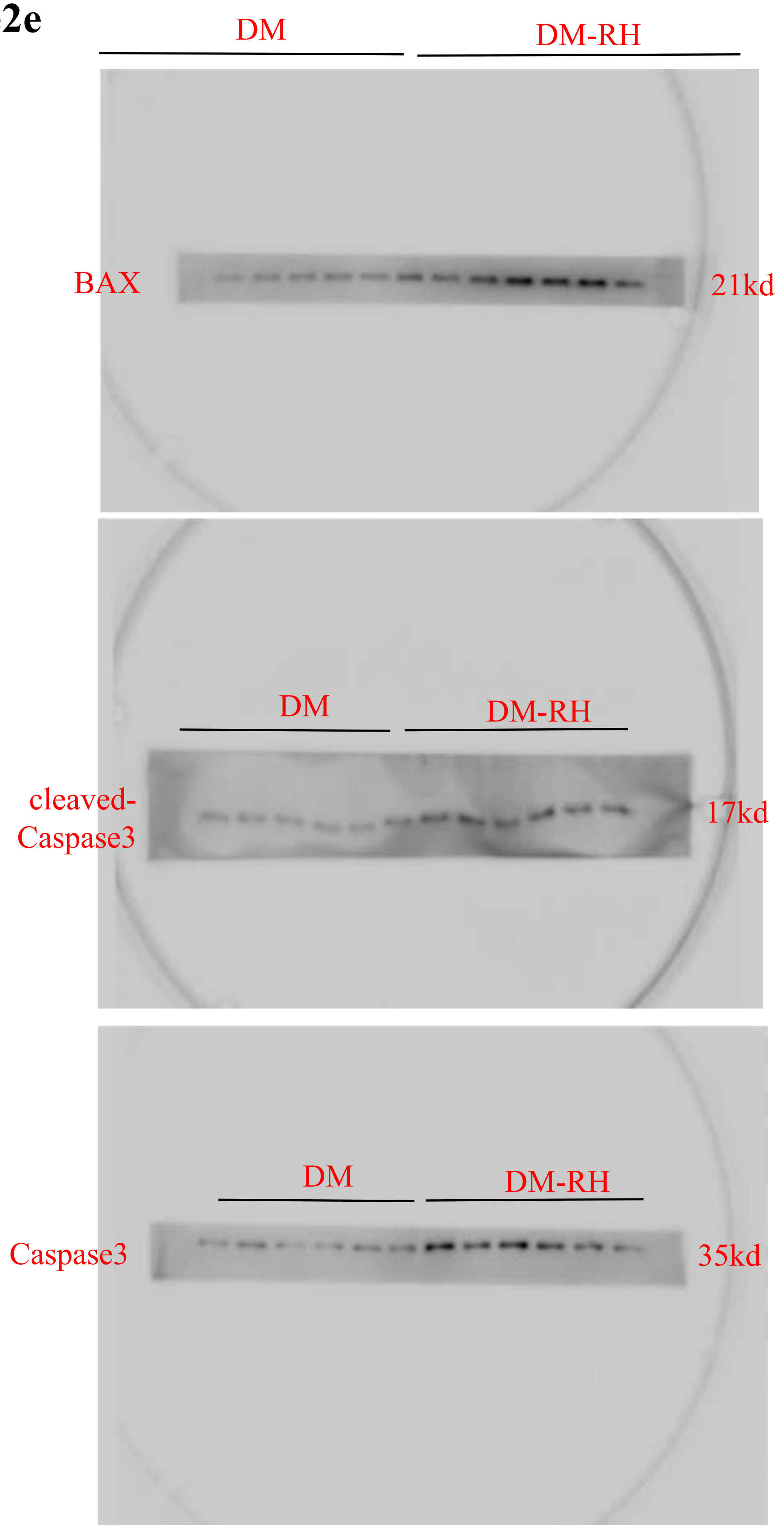
DM
DM-RH
BAX
21kd
DM
DM-RH
cleaved-
Caspase3
17kd
DM
DM-RH
Caspase3
35kd

Figure2e

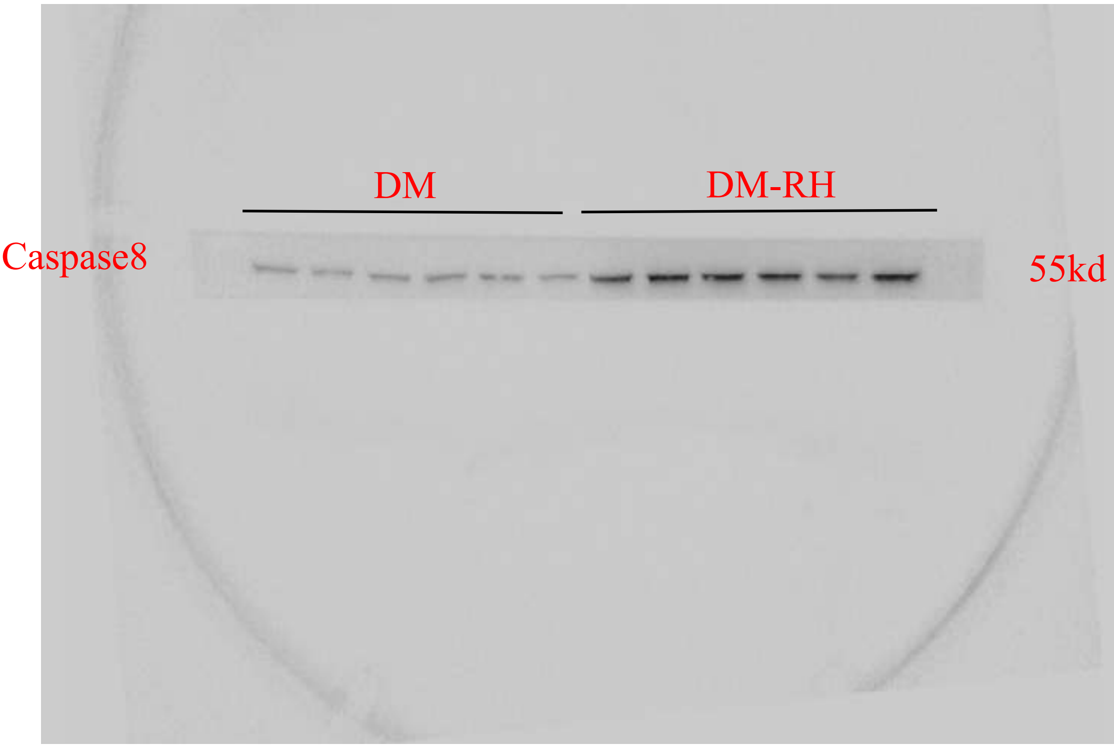

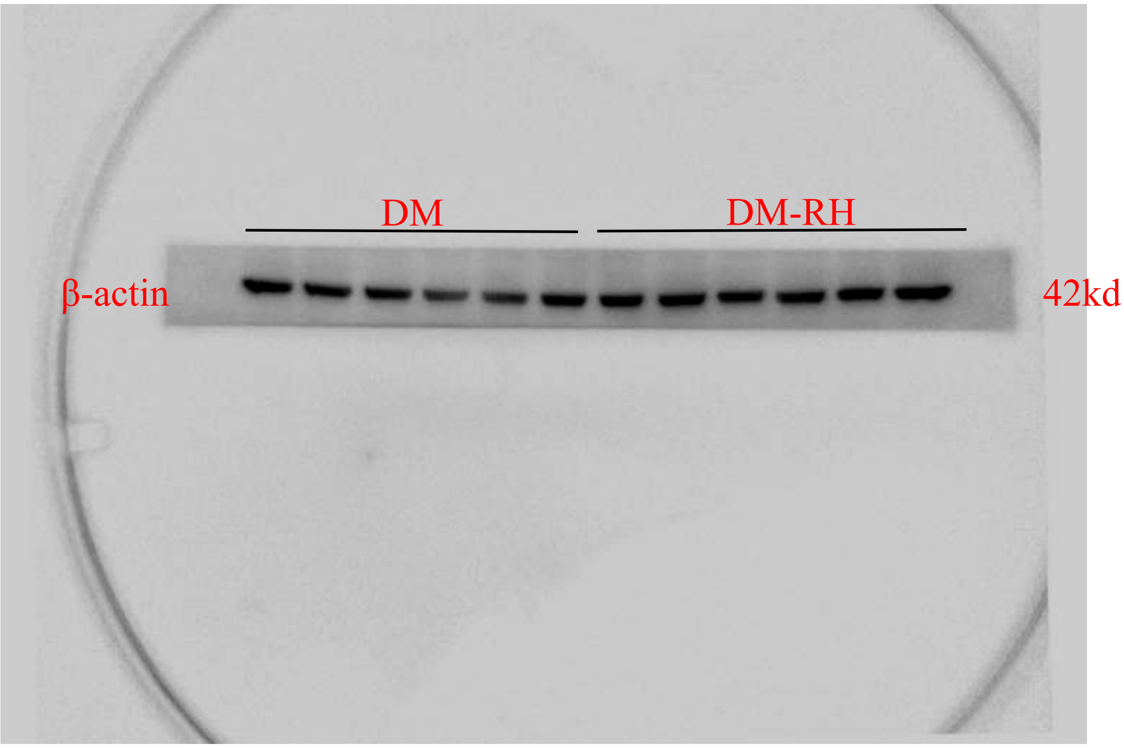

Figure2f

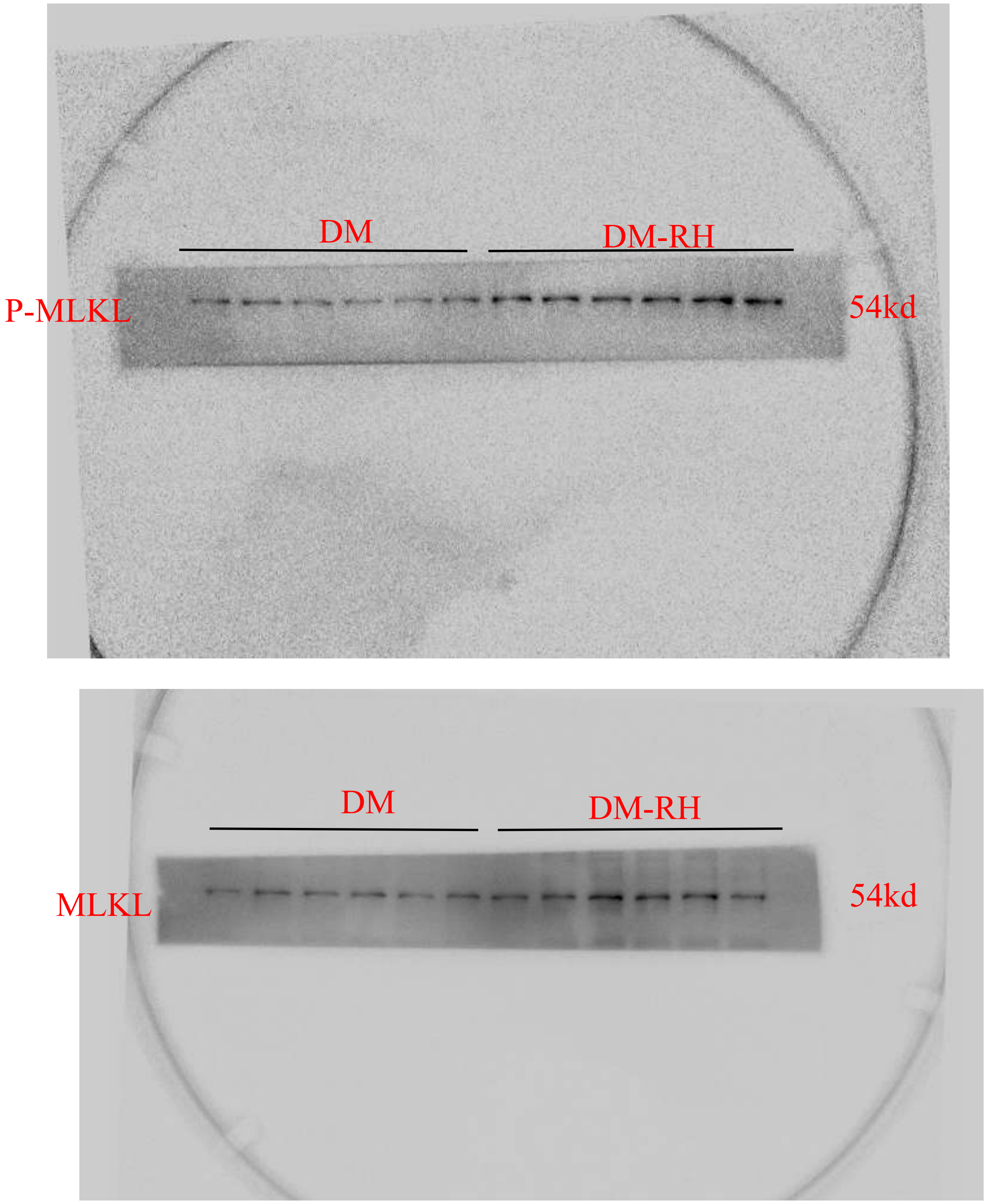

Figure2f

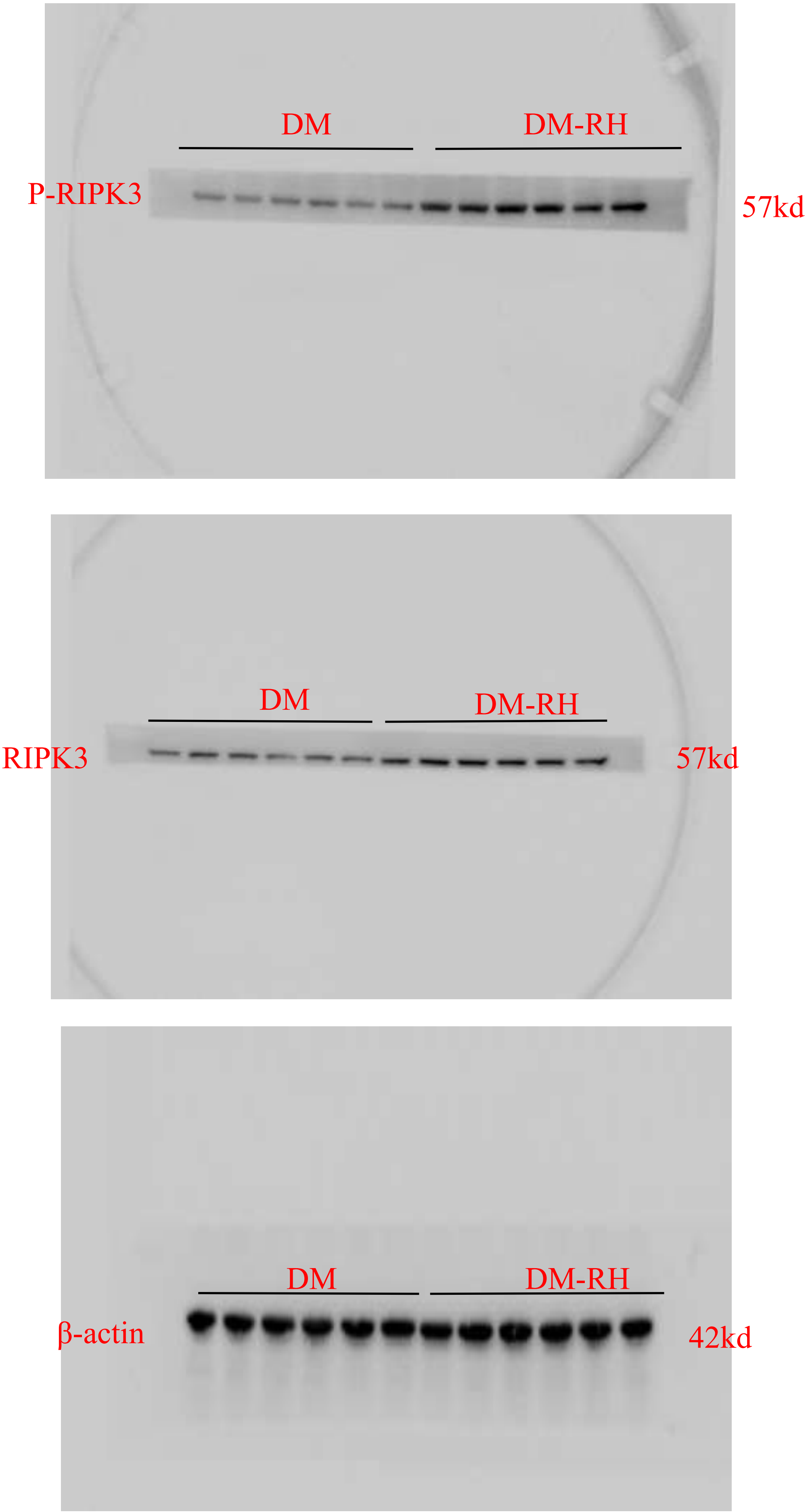

Figure2h

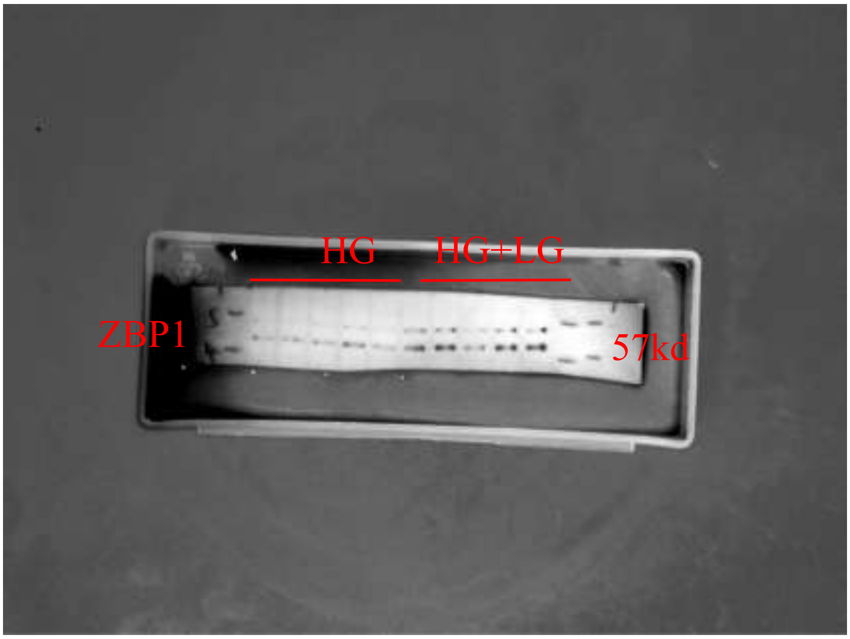

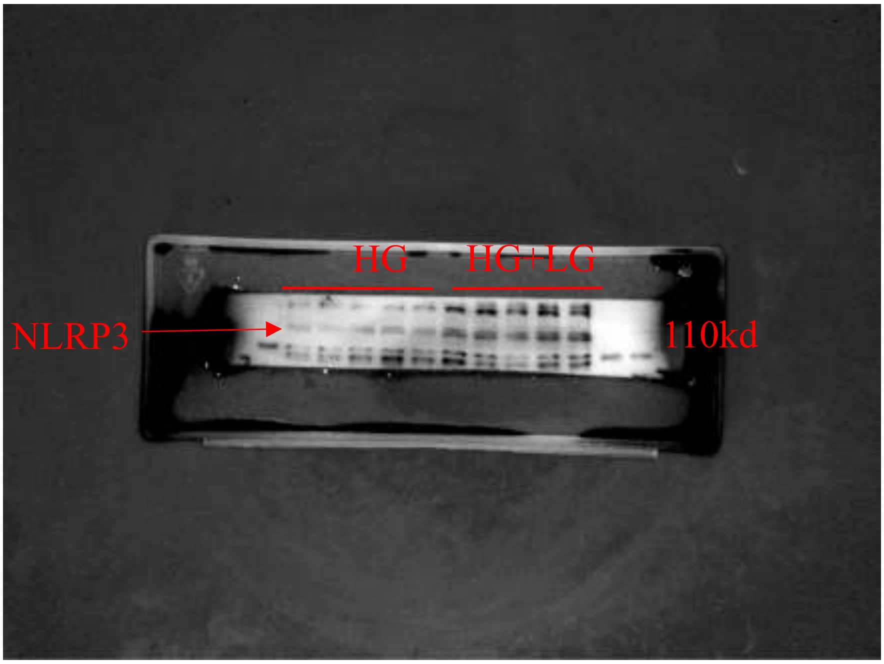

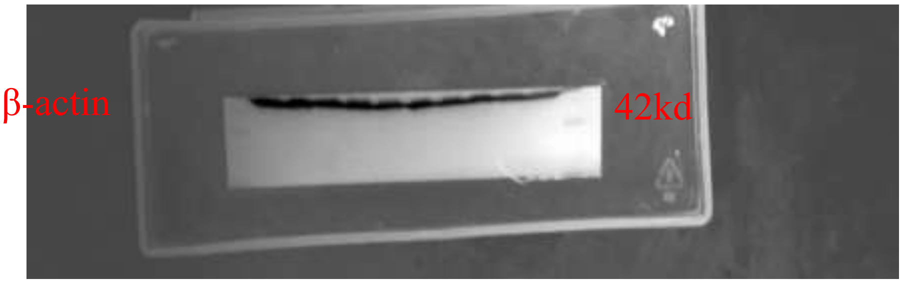

Figure2h

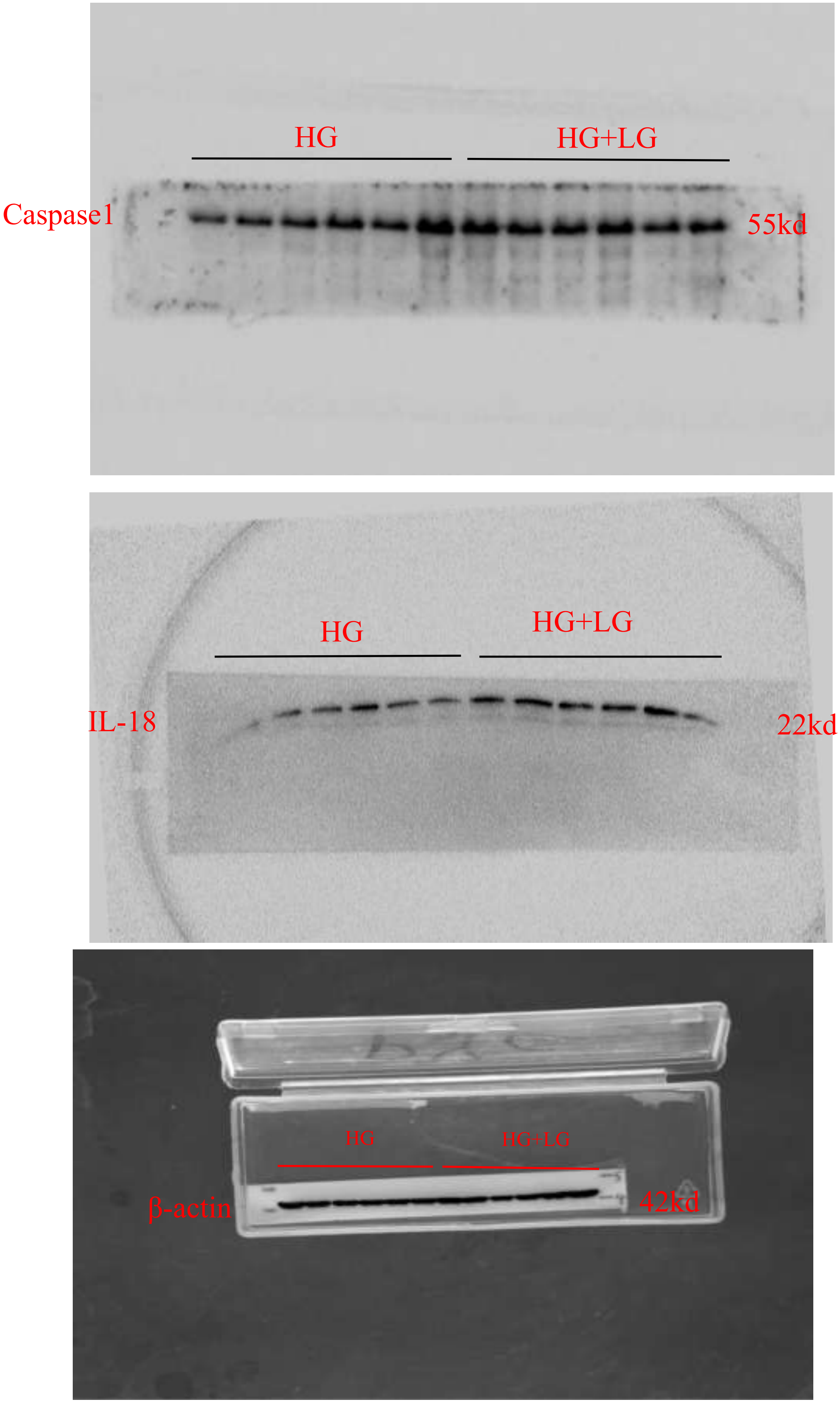

Figure2i

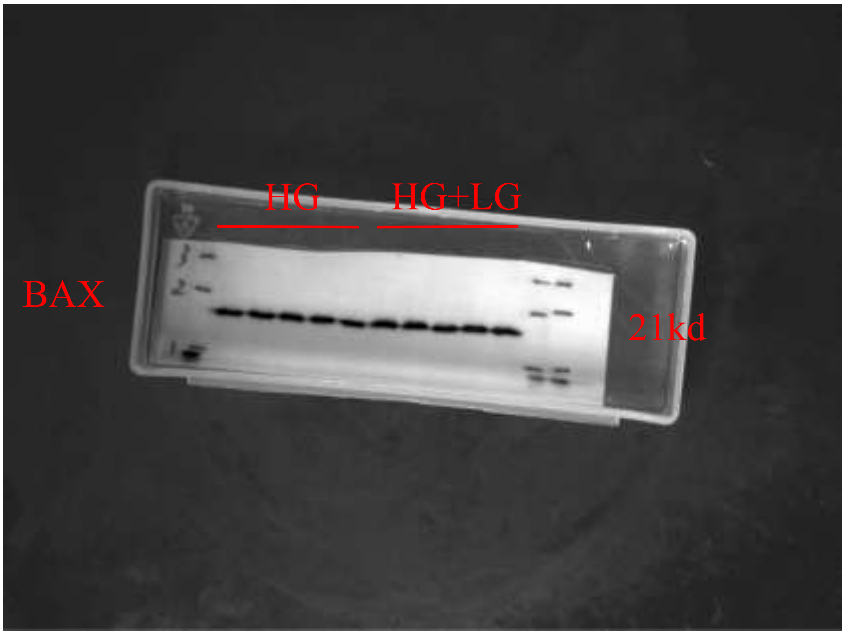

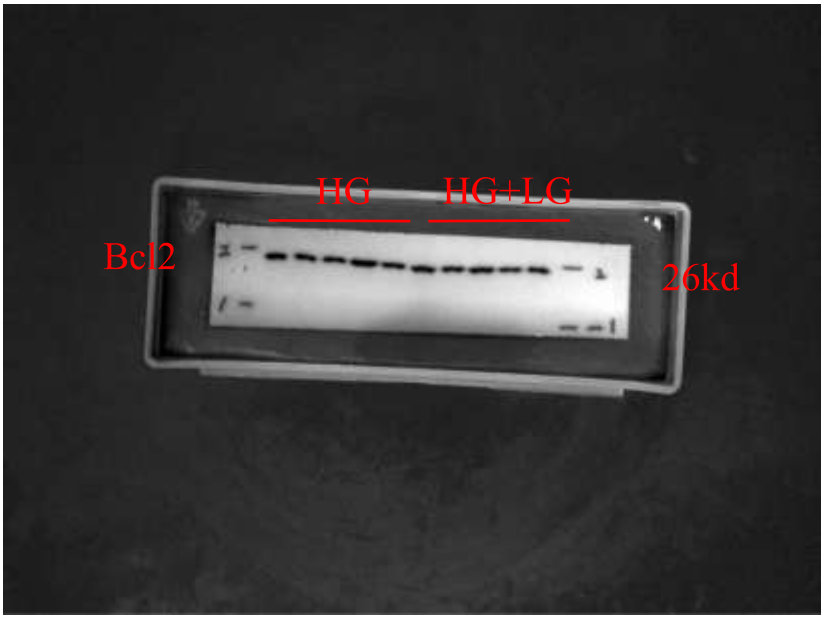

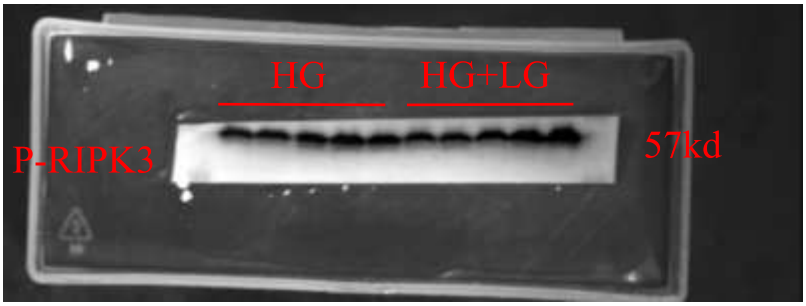

Figure2i

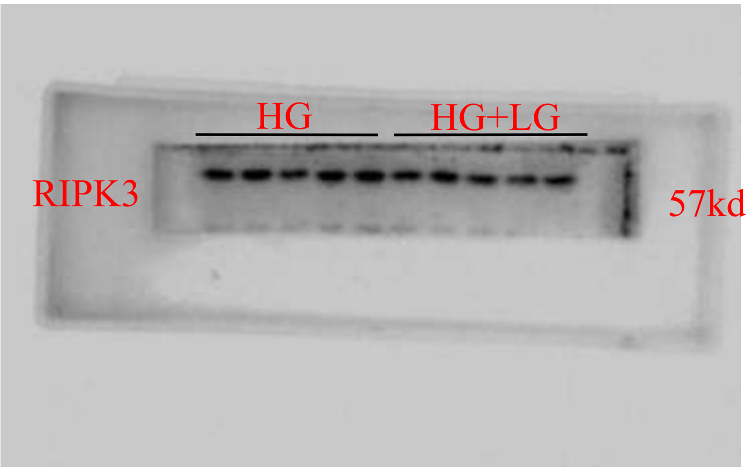

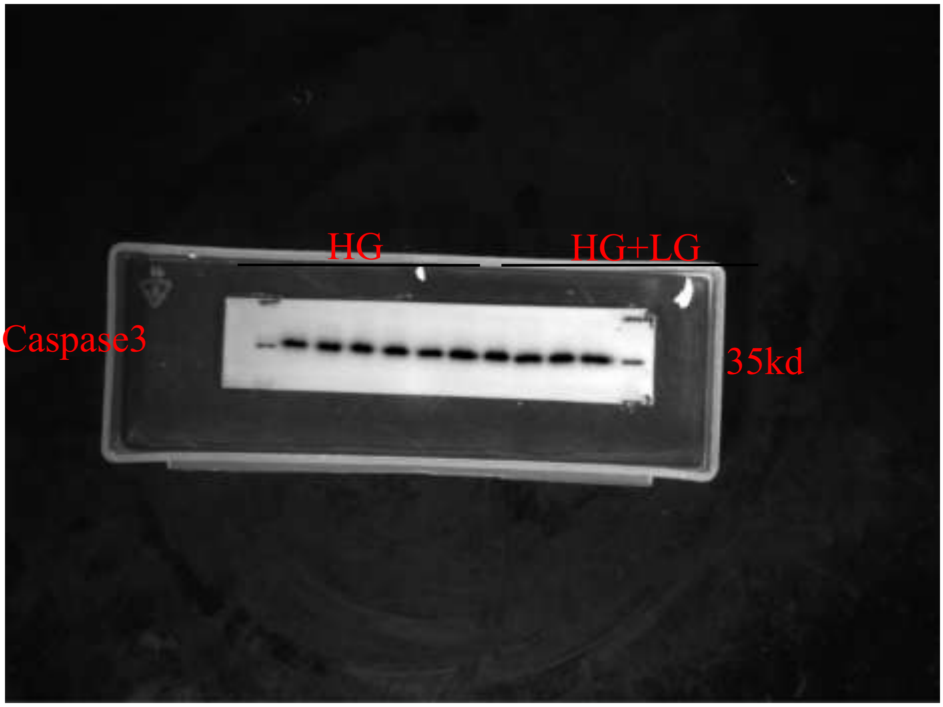

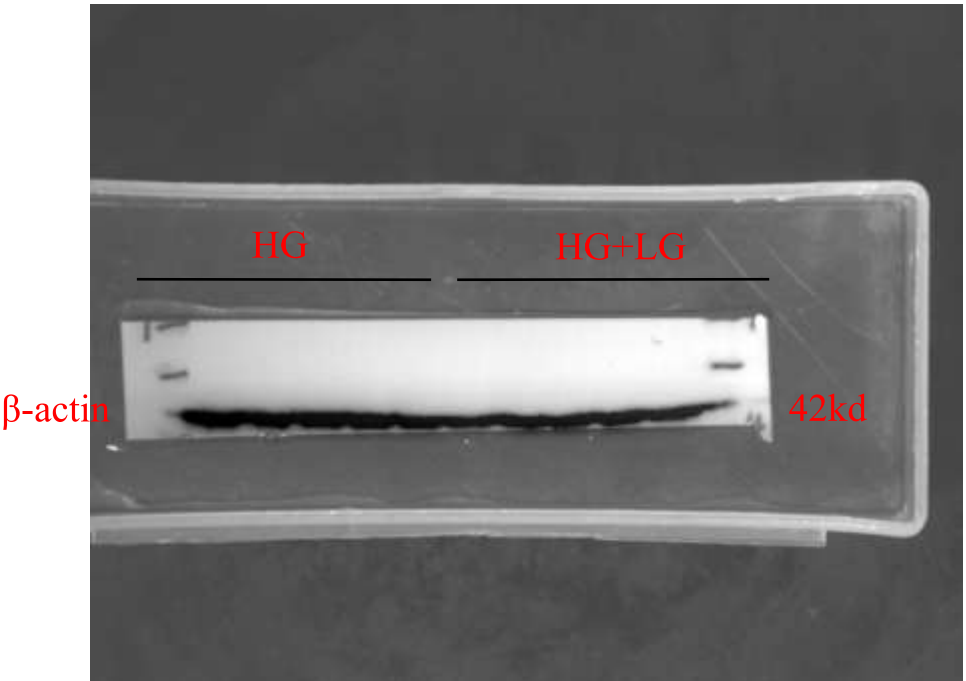

Figure3a

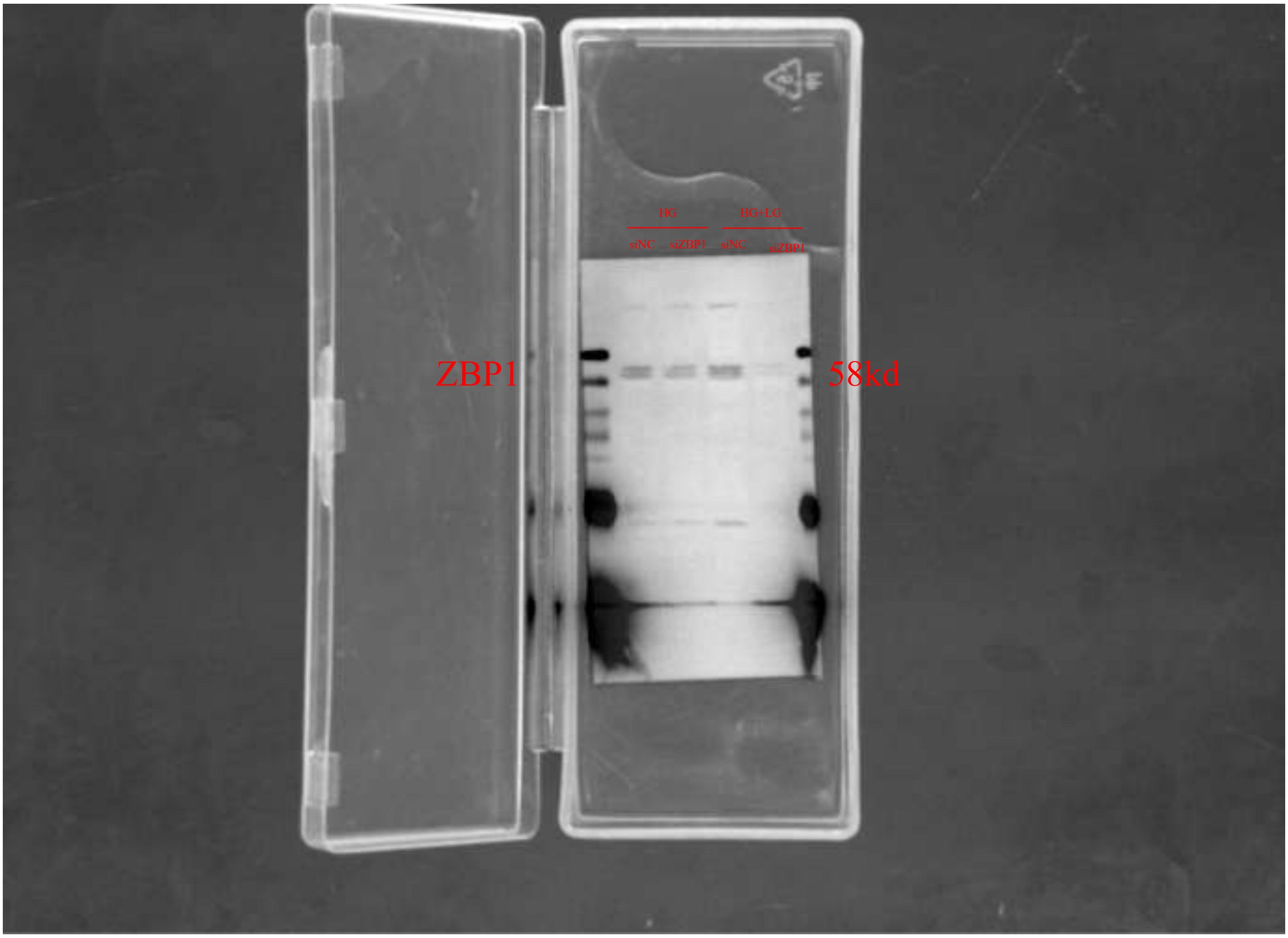

Figure3a

NLRP12

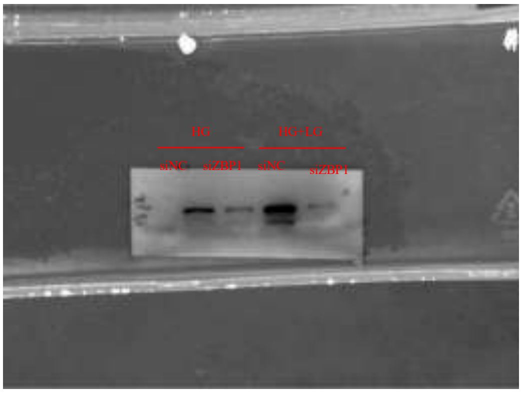

110kd

P-RIPK1

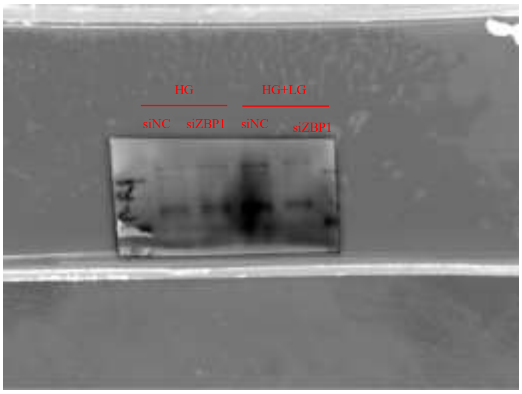

76kd

RIPK1

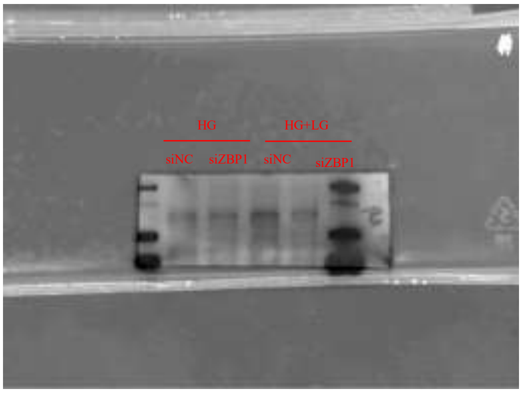

76kd

Figure3a

P-MLKL

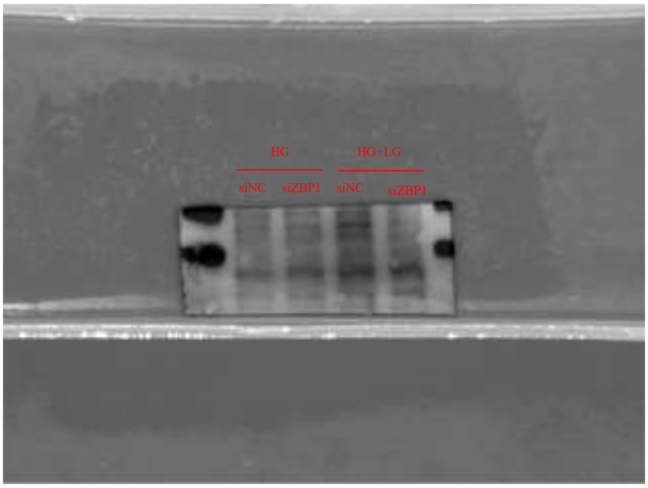

55kd

MLKL

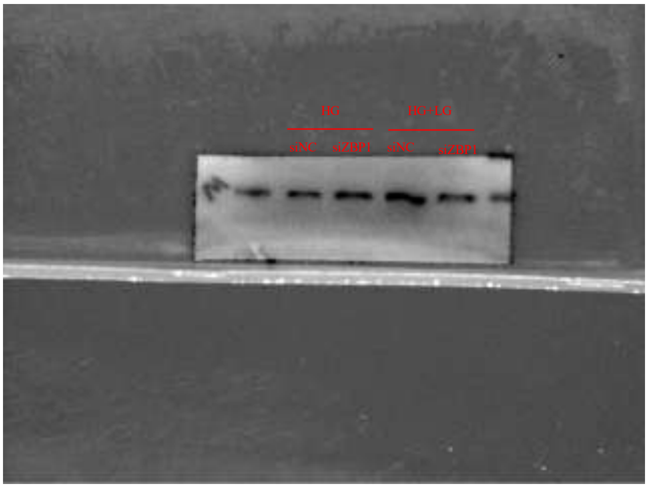

55kd

β-actin

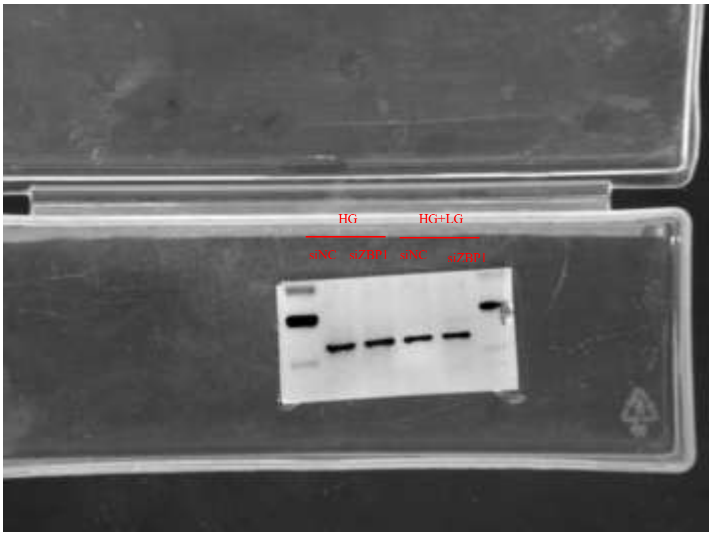

42kd

Figure3b

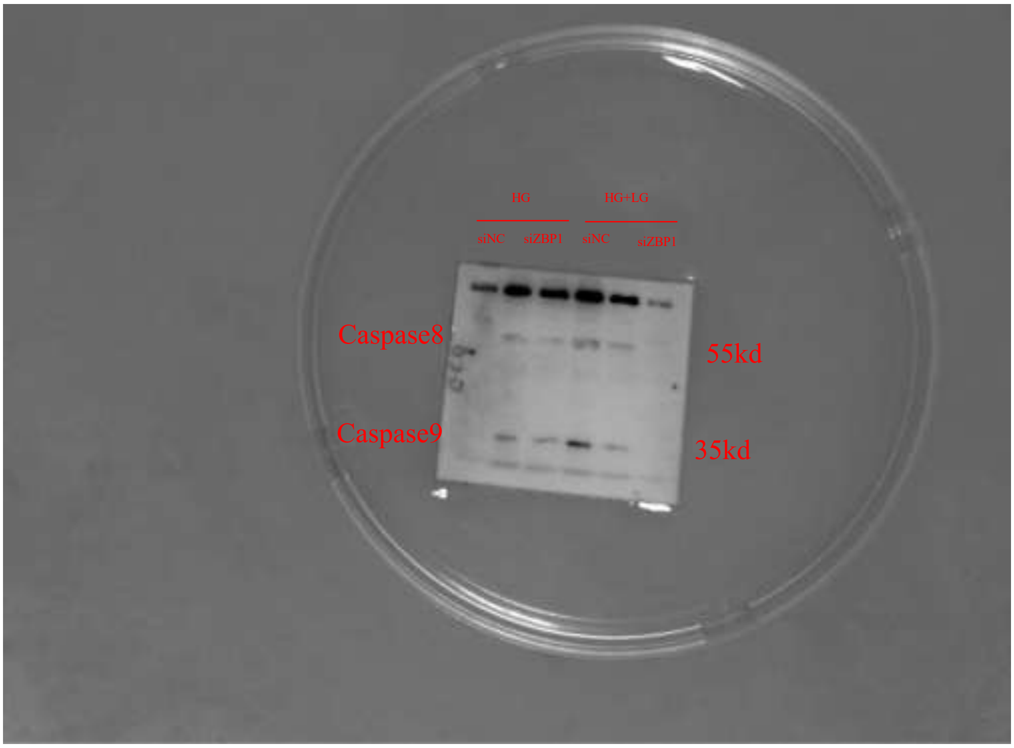
HG
HG+LG
siNC
siZBP1
siNC
siZBP1
Caspase8
55kd
Caspase9
35kd

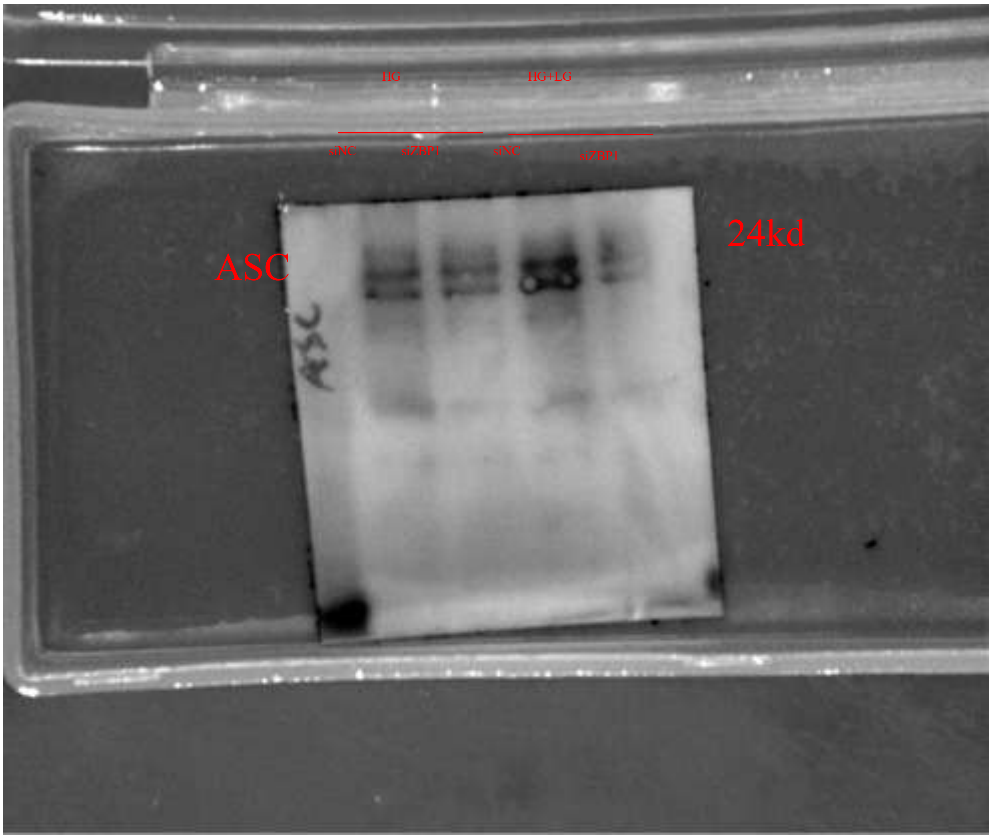
HG
HG+LG
siNC
siZBP1
siNC
siZBP1
ASC
24kd

Figure3b

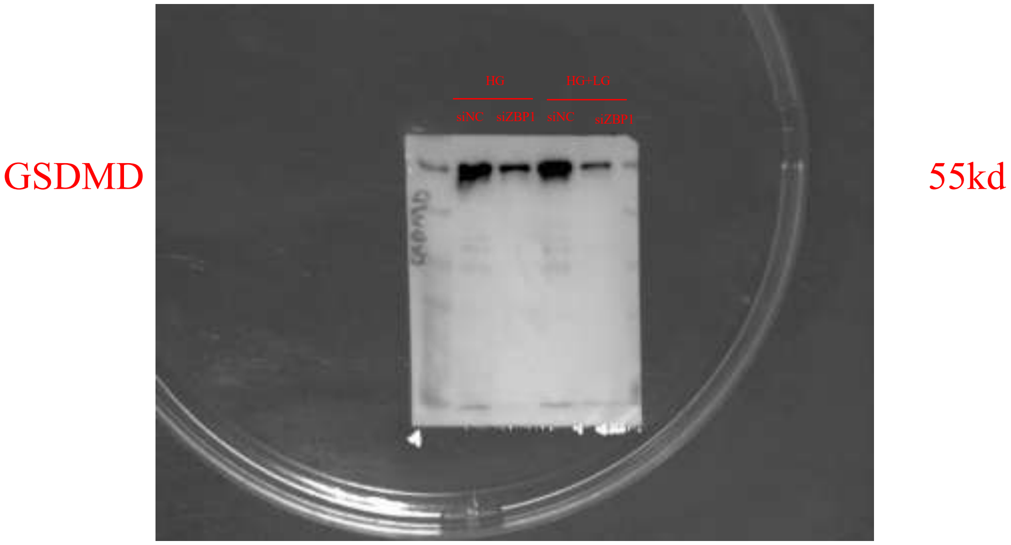
GSDMD
55kd
HG
HG+LG
siNC
siZBP1
siNC
siZBP1

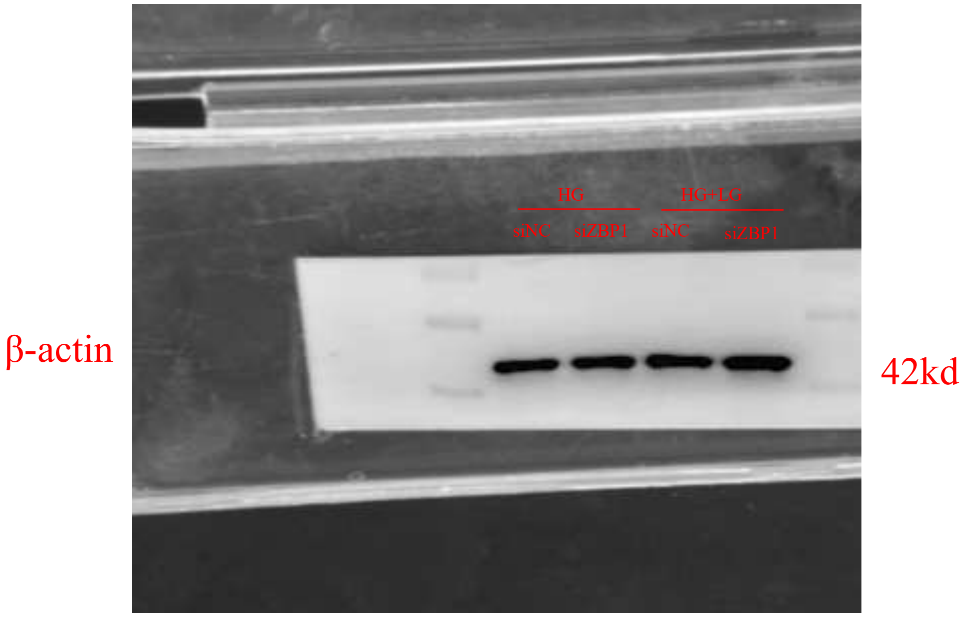
β-actin
42kd
HG
HG+LG
siNC
siZBP1
siNC
siZBP1

Figure4a

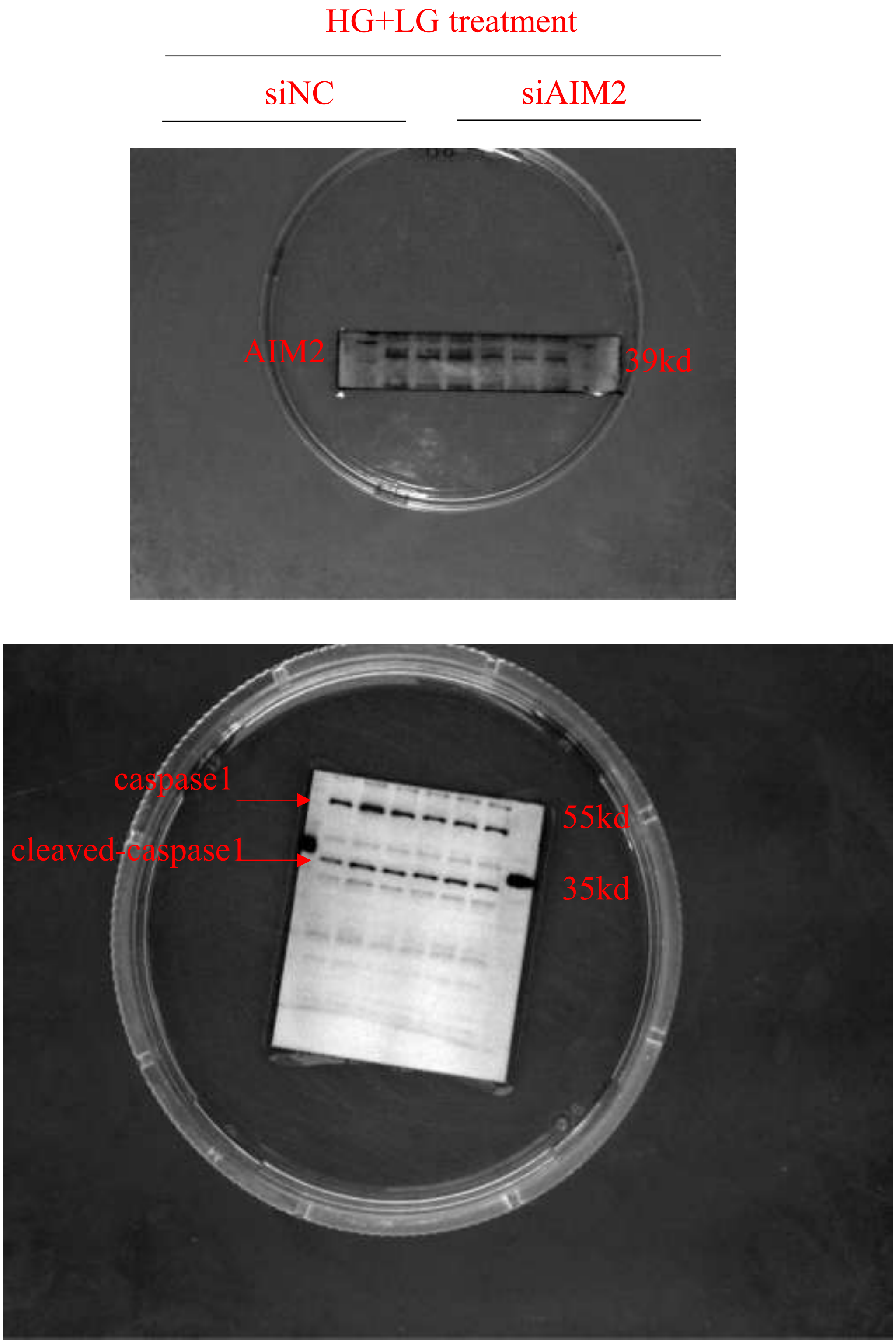
HG+LG treatment
siNC
siAIM2
AIM2
39kd
caspase1
cleaved-caspase1
55kd
35kd

Figure4a

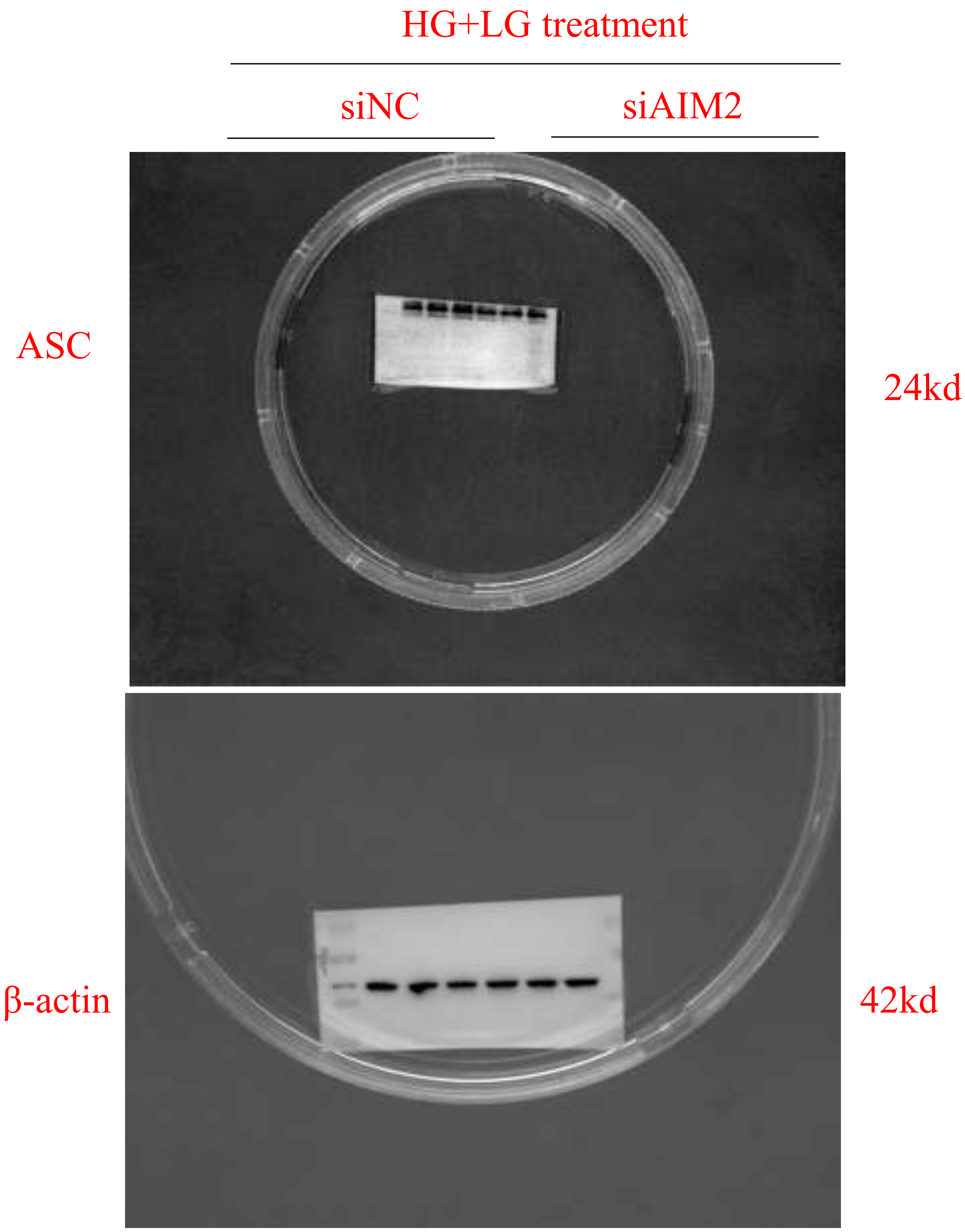

Figure4b

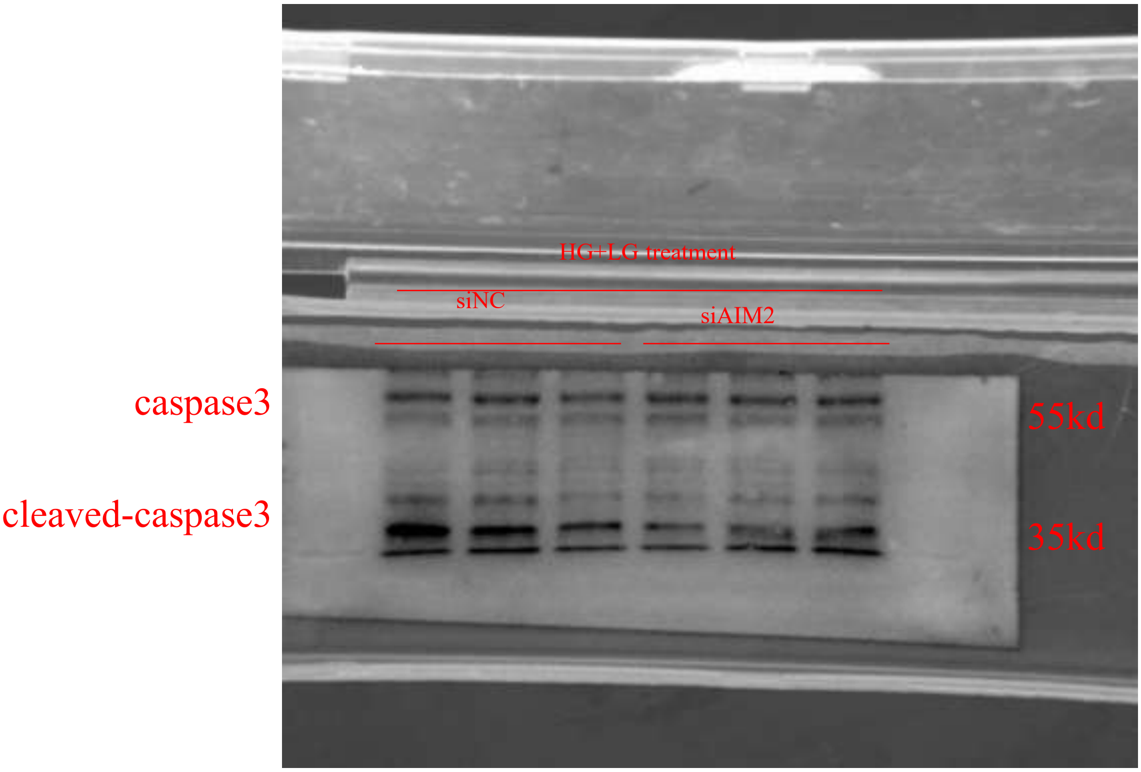

HG+LG treatment
siNC
siAIM2
caspase3
cleaved-caspase3
55kd
35kd

Figure4b

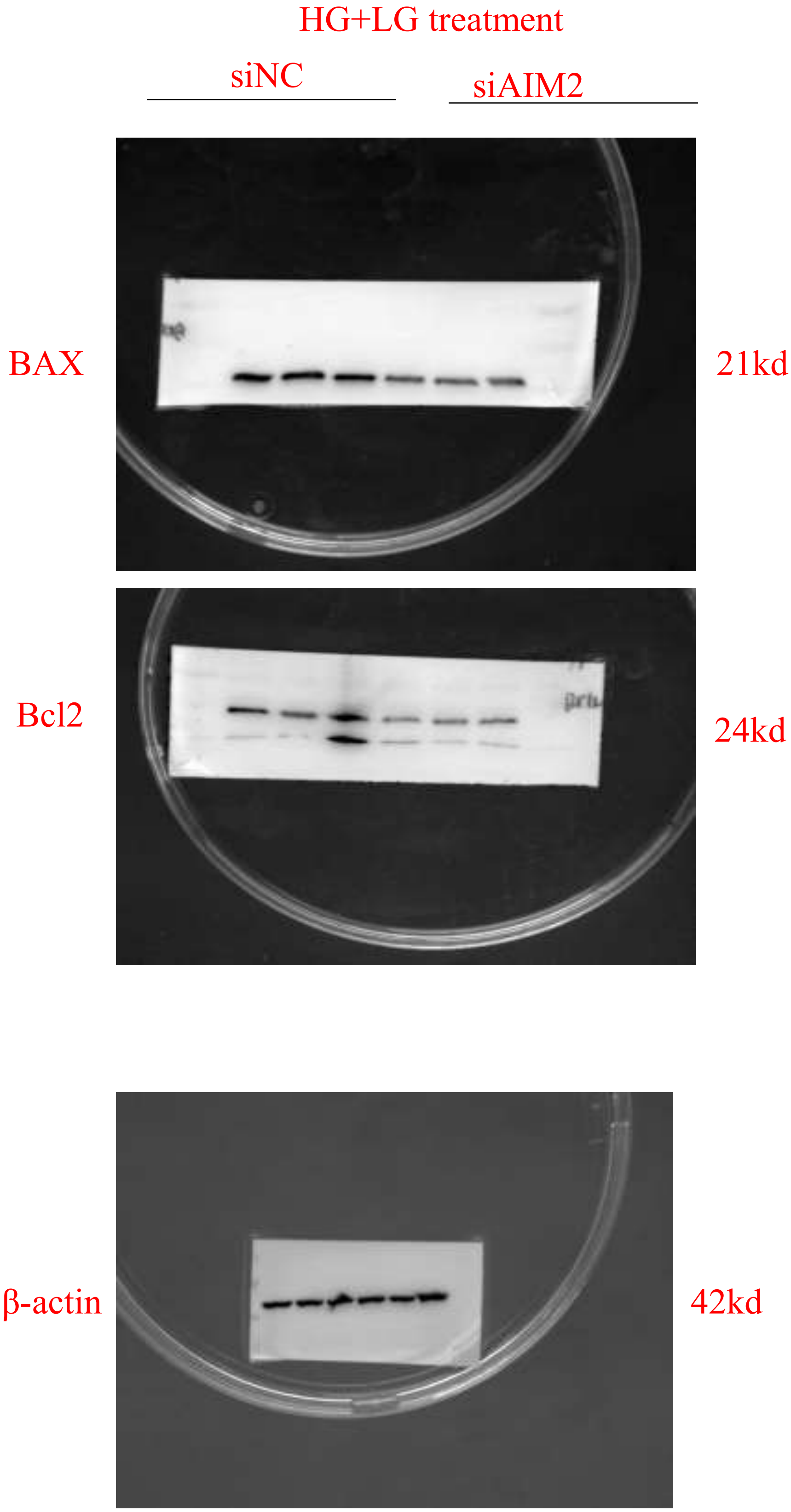

Figure4c

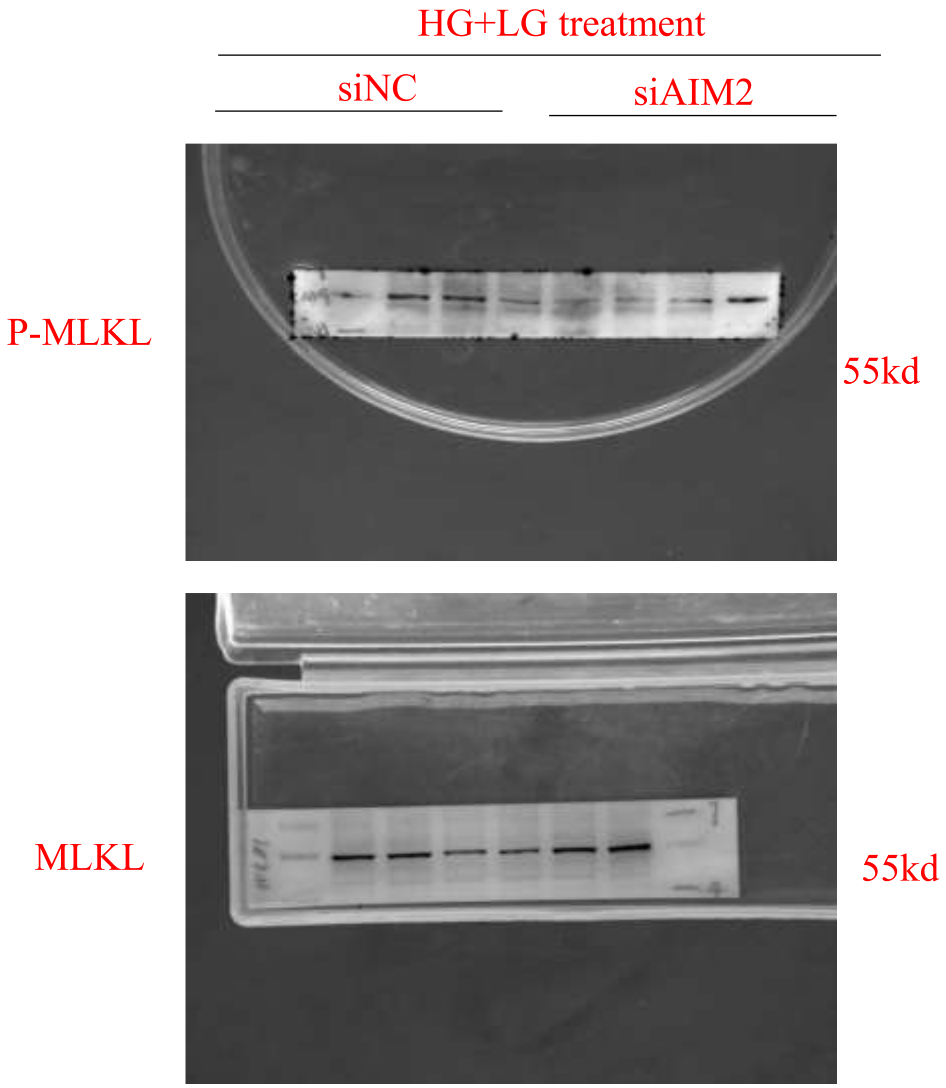

Figure4c

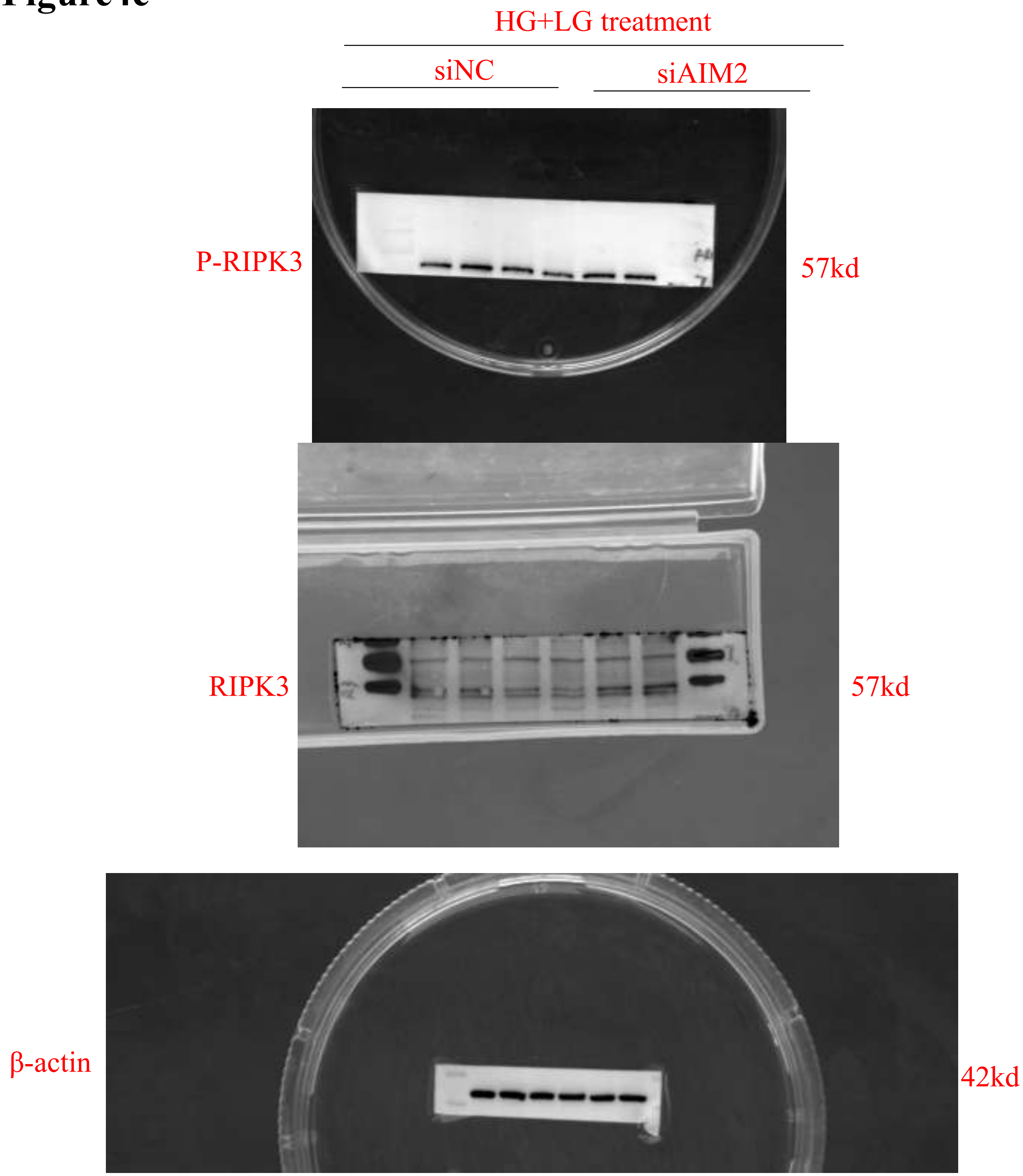
HG+LG treatment
siNC
siAIM2
P-RIPK3
57kd
RIPK3
57kd
β-actin
42kd

Figure4d

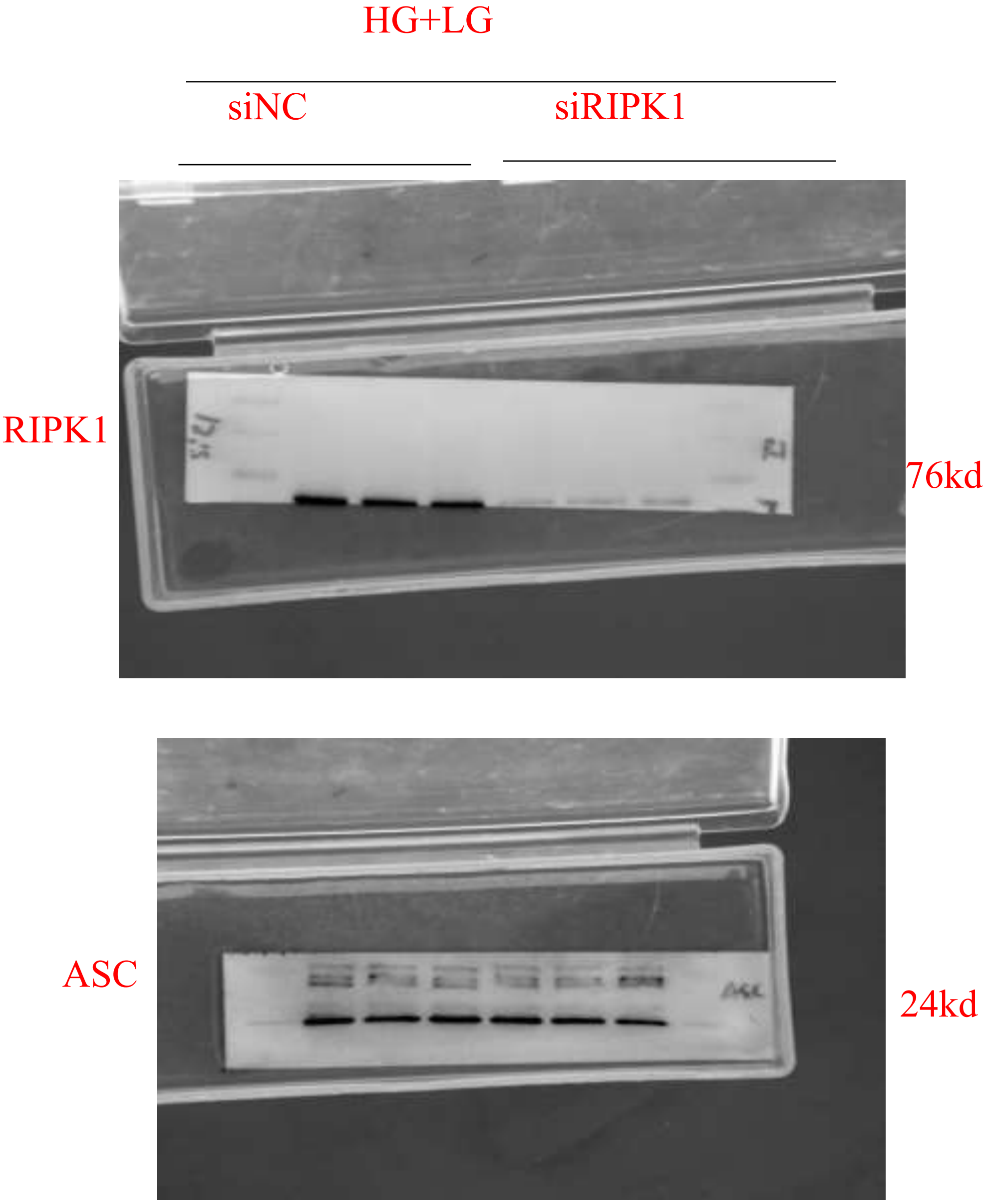

Figure4d

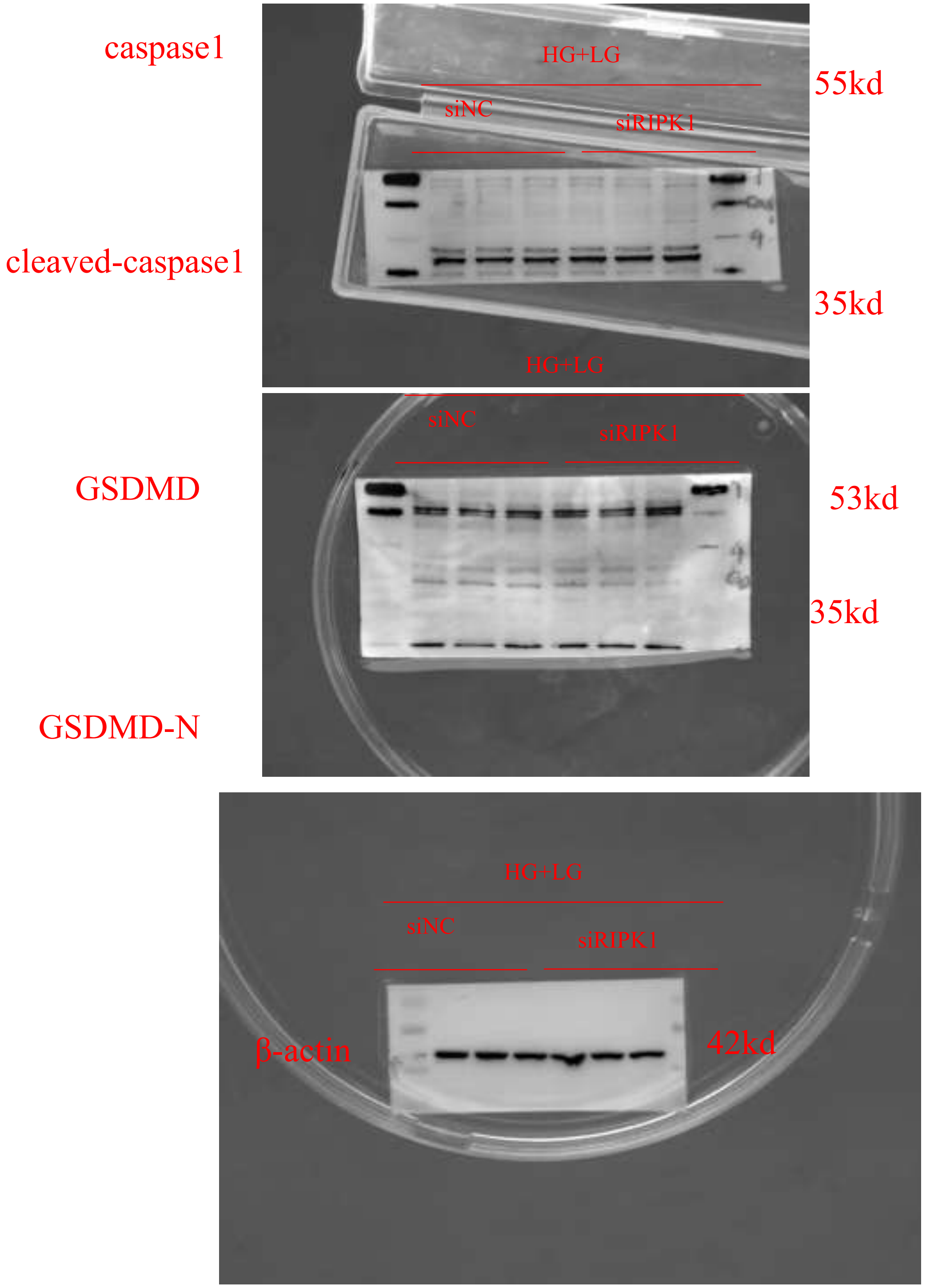
caspase1
HG+LG
siNC
siRIPK1
55kd
cleaved-caspase1
35kd
HG+LG
siNC
siRIPK1
GSDMD
53kd
35kd
GSDMD-N
HG+LG
siNC
siRIPK1
β-actin
42kd

Figure4e

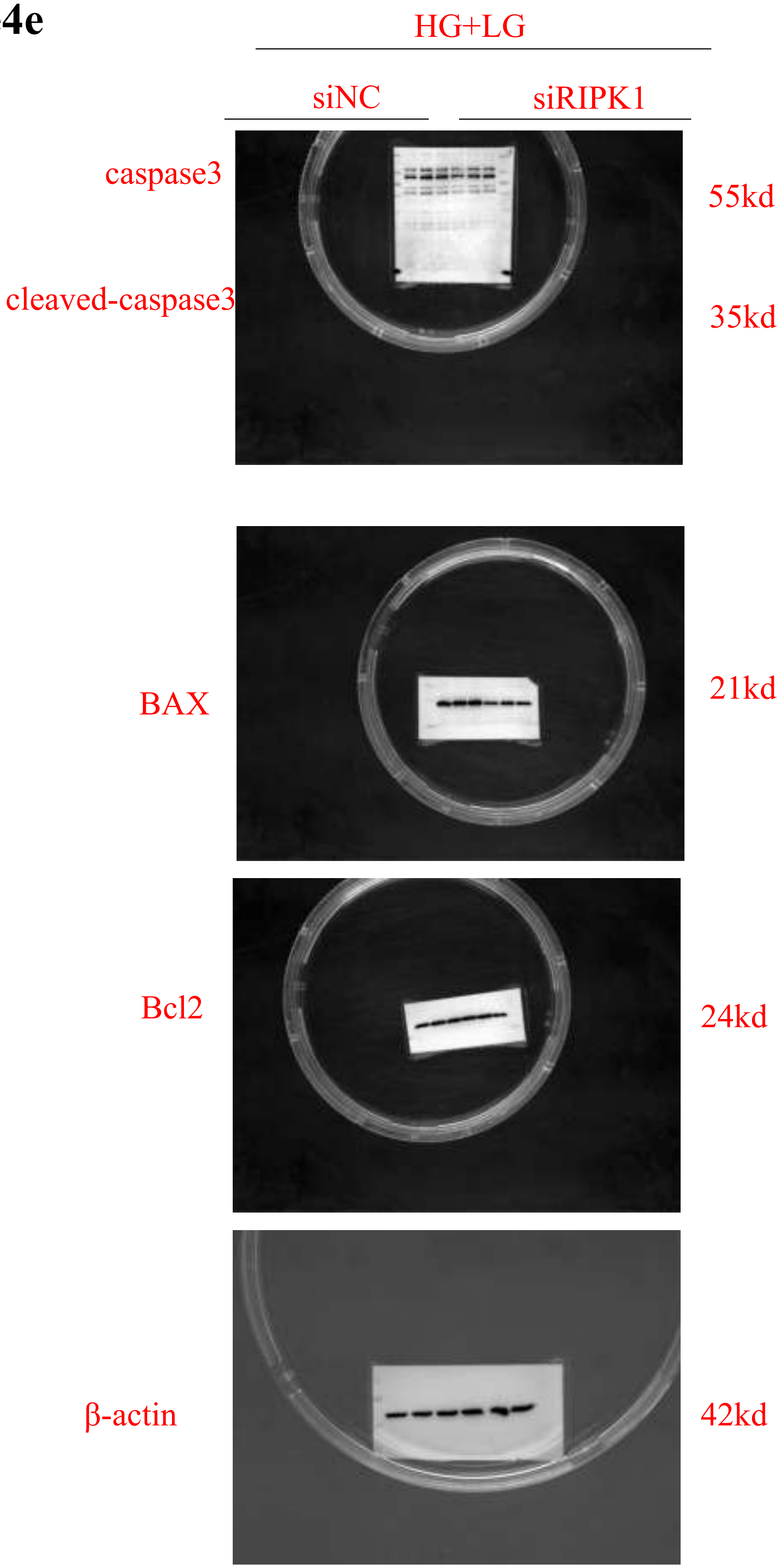
HG+LG
siNC
siRIPK1
caspase3
cleaved-caspase3
55kd
35kd
BAX
21kd
Bcl2
24kd
β-actin
42kd

Figure4f

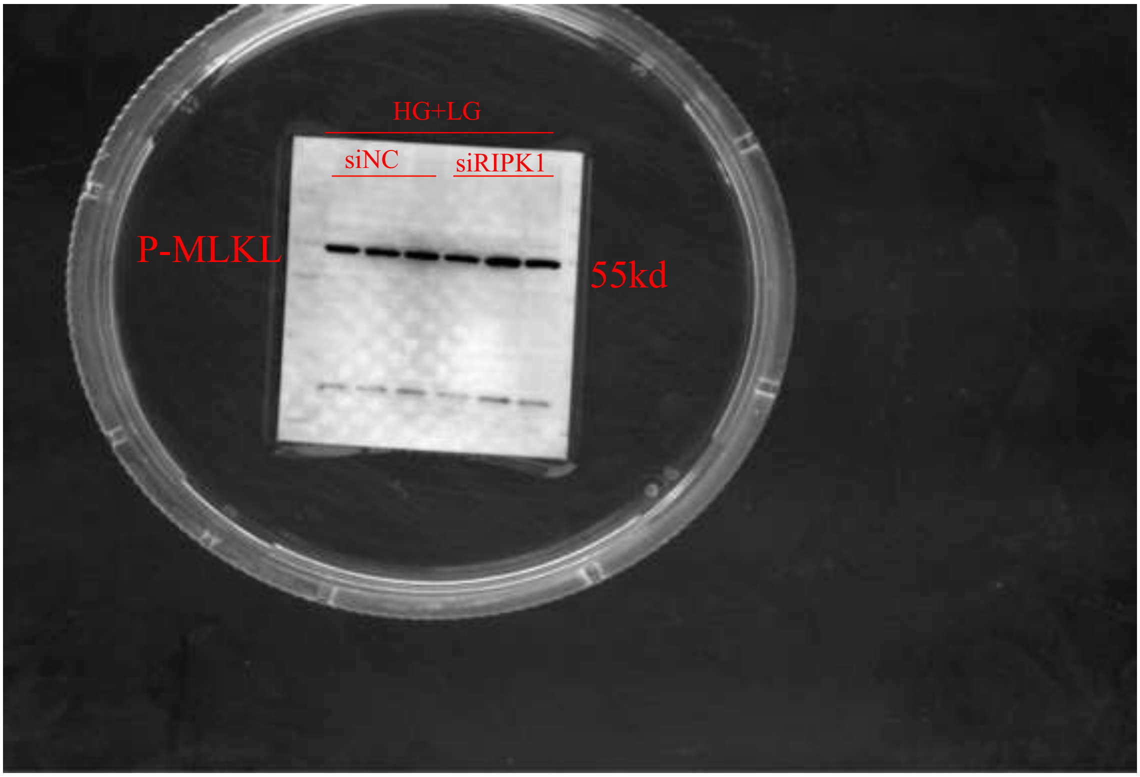

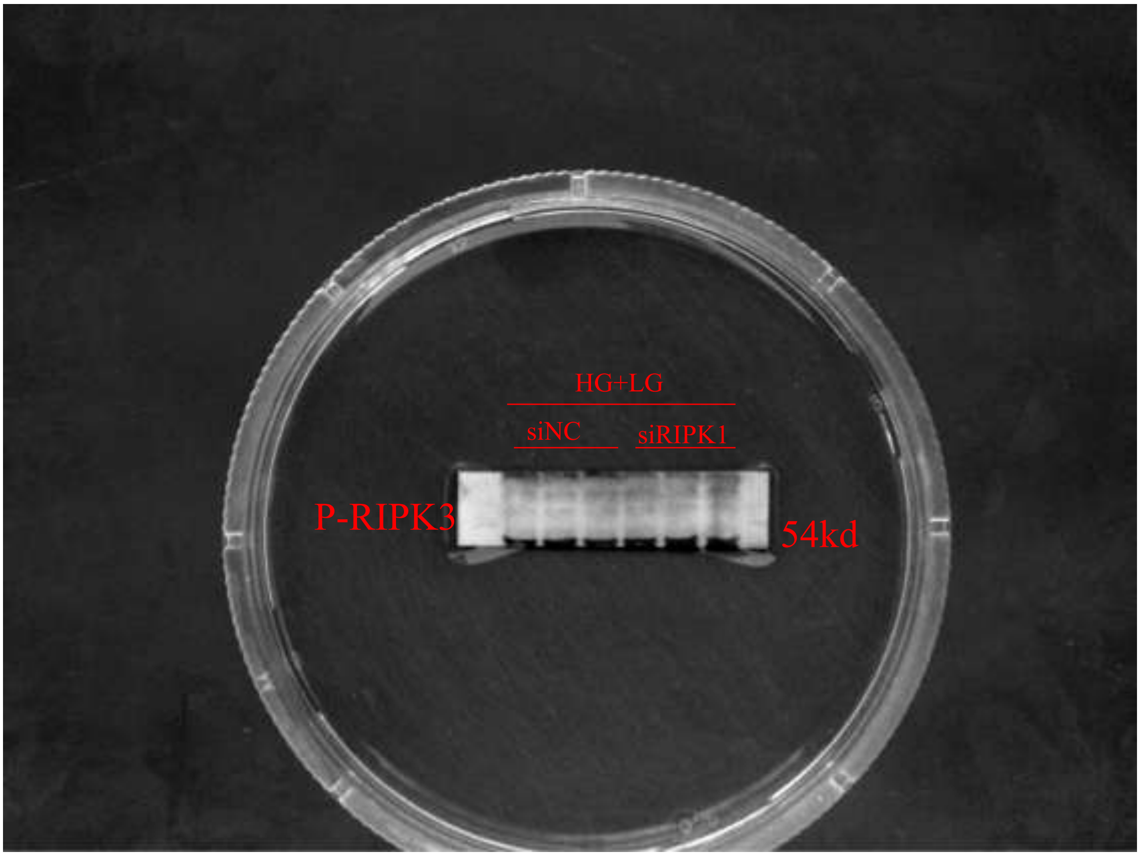

Figure4f

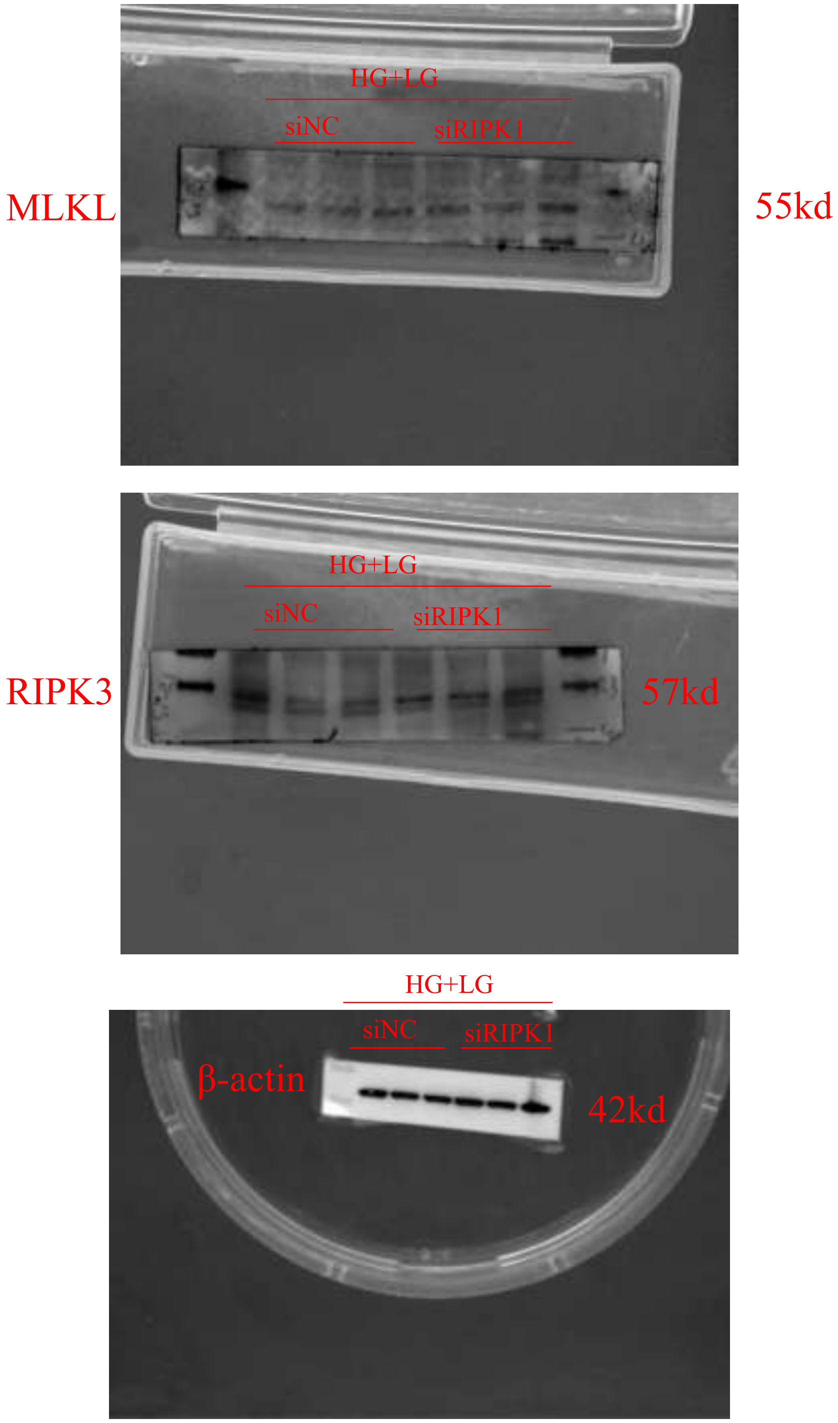
HG+LG
siNC
siRIPK1
MLKL
55kd
HG+LG
siNC
siRIPK1
RIPK3
57kd
HG+LG
siNC
siRIPK1
β-actin
42kd

Figure5e

RAGE

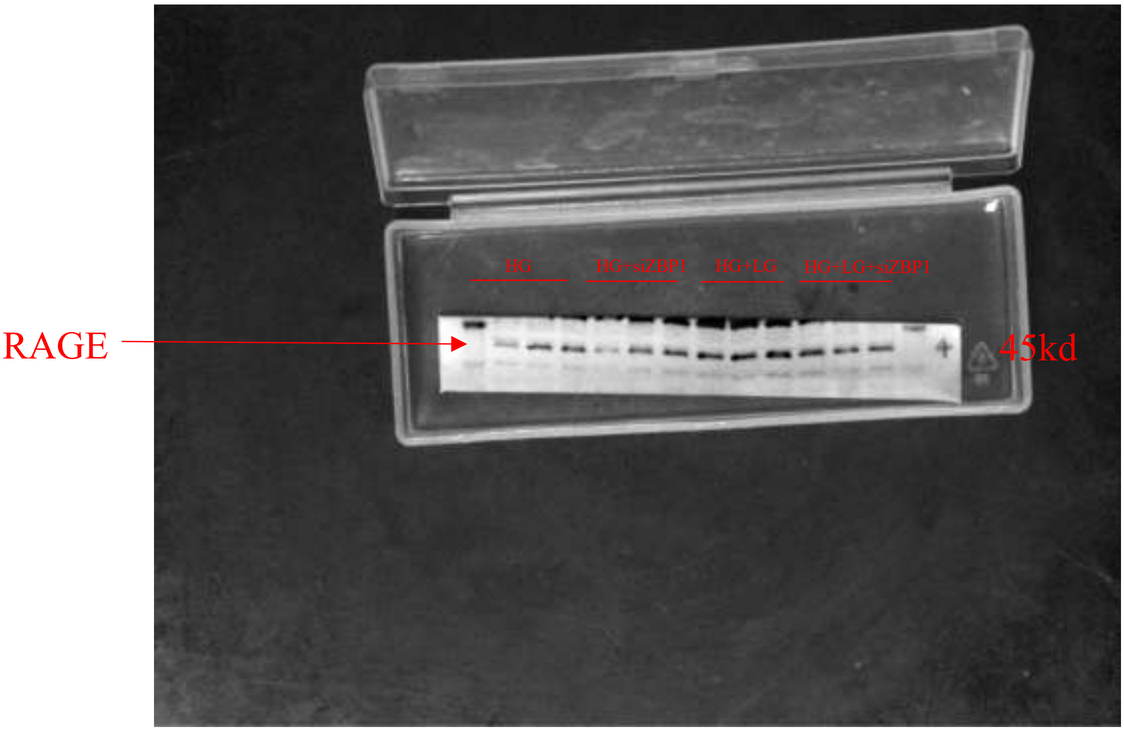

β-actin

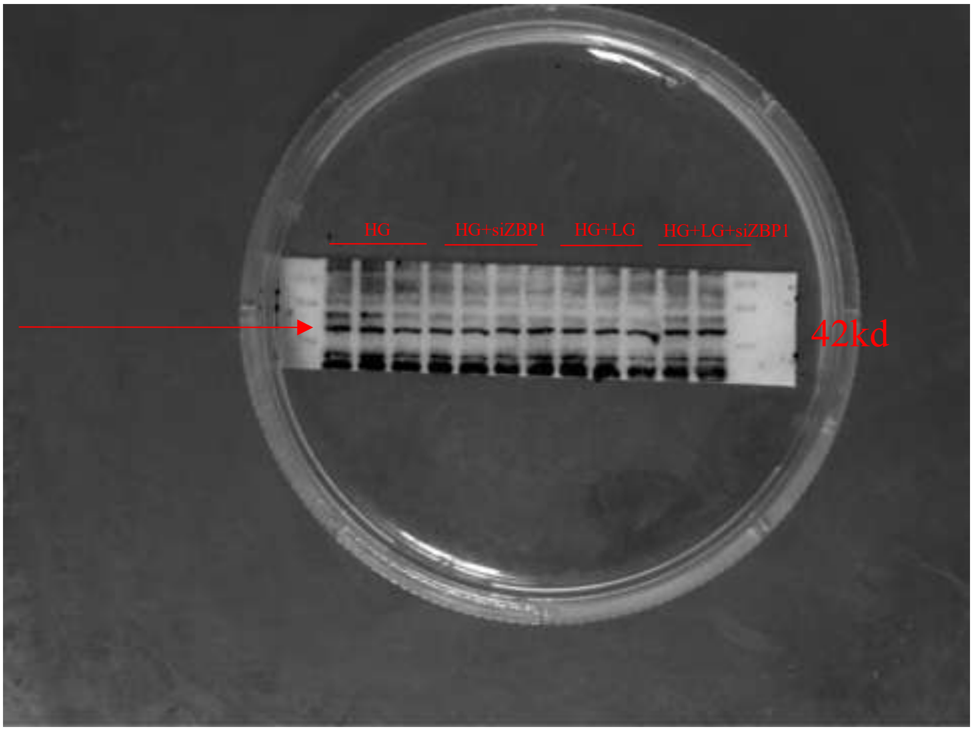

Figure5i

RAGE

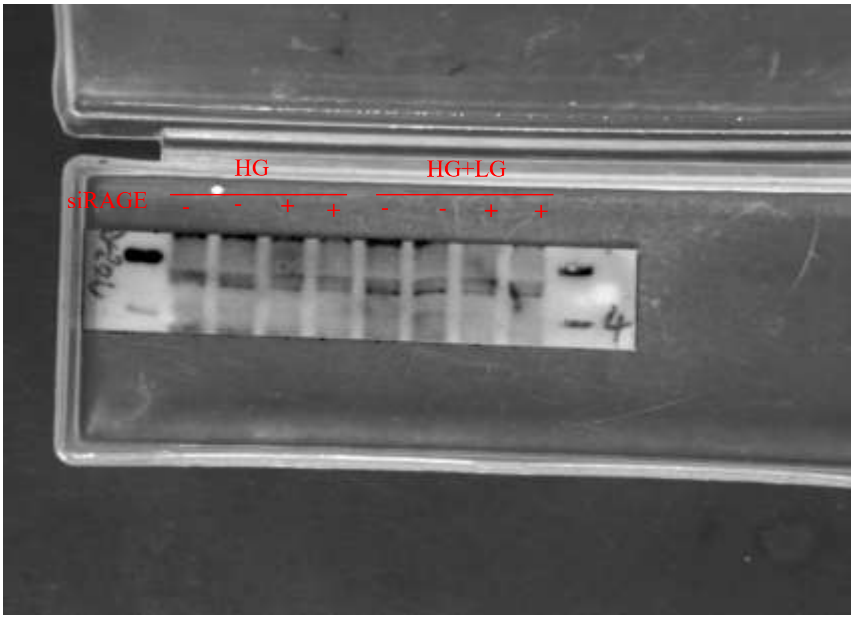

45kd

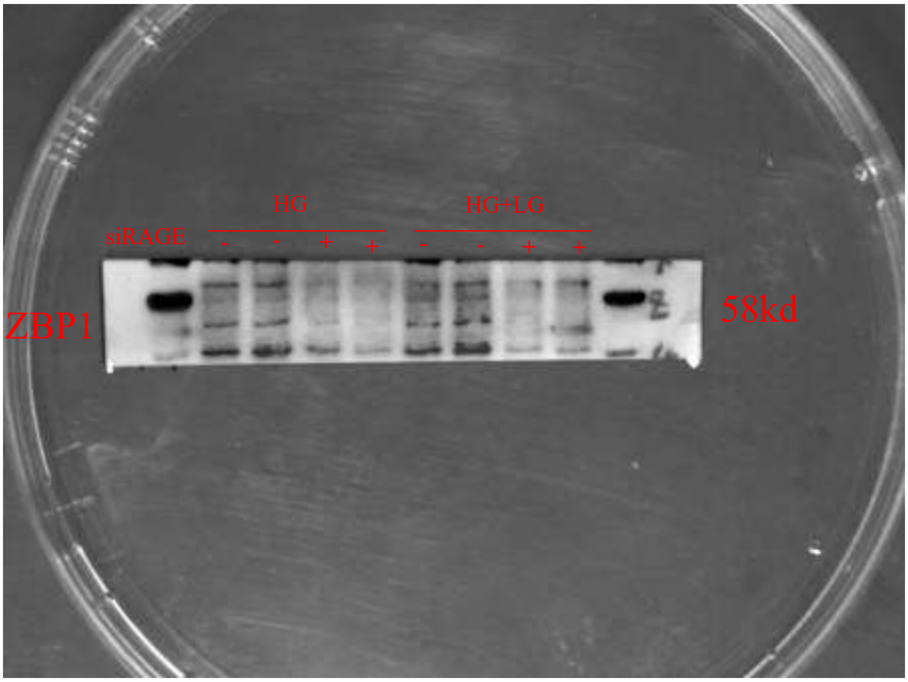
HG
HG+LG
siRAGE
- - + + - - + +
ZBP1
58kd

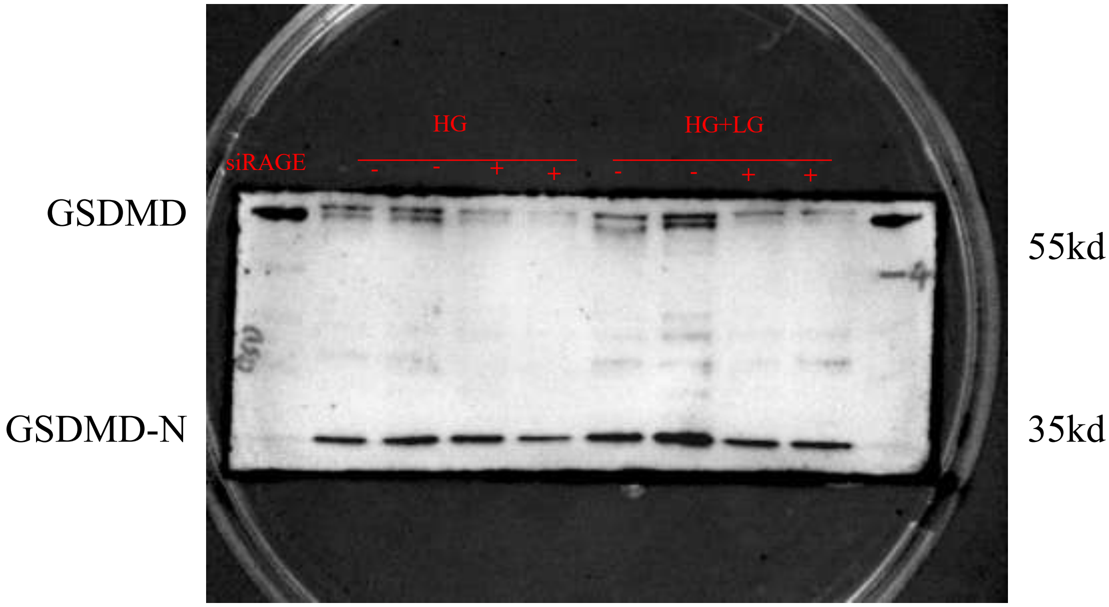
HG
HG+LG
siRAGE
-
-
+
+
-
-
+
+
GSDMD
GSDMD-N
55kd
35kd

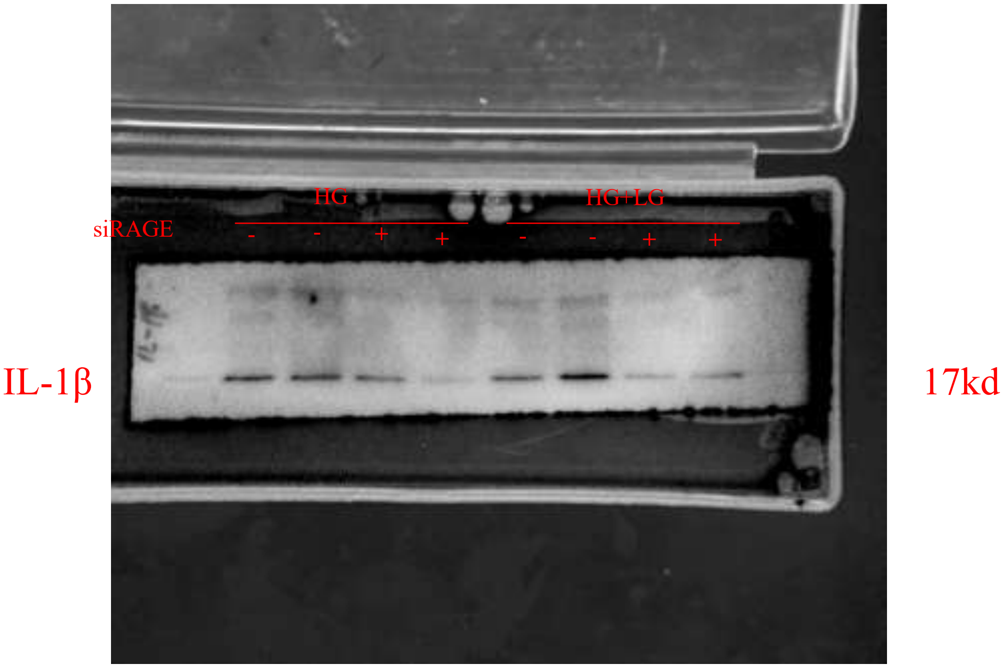
HG
HG+LG
siRAGE
- - + + - - + +
IL-1β
17kd

Figure5i

BAX

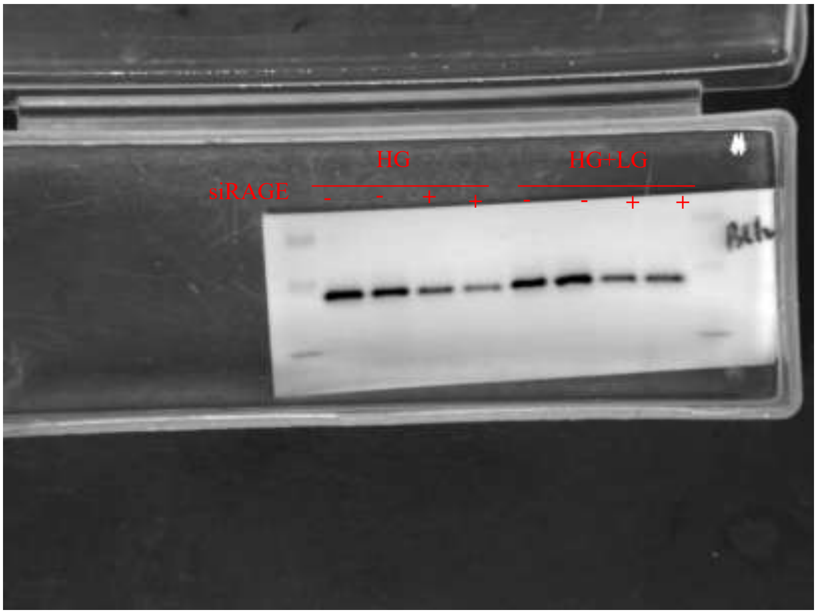

21kd

Figure5i

Bcl2

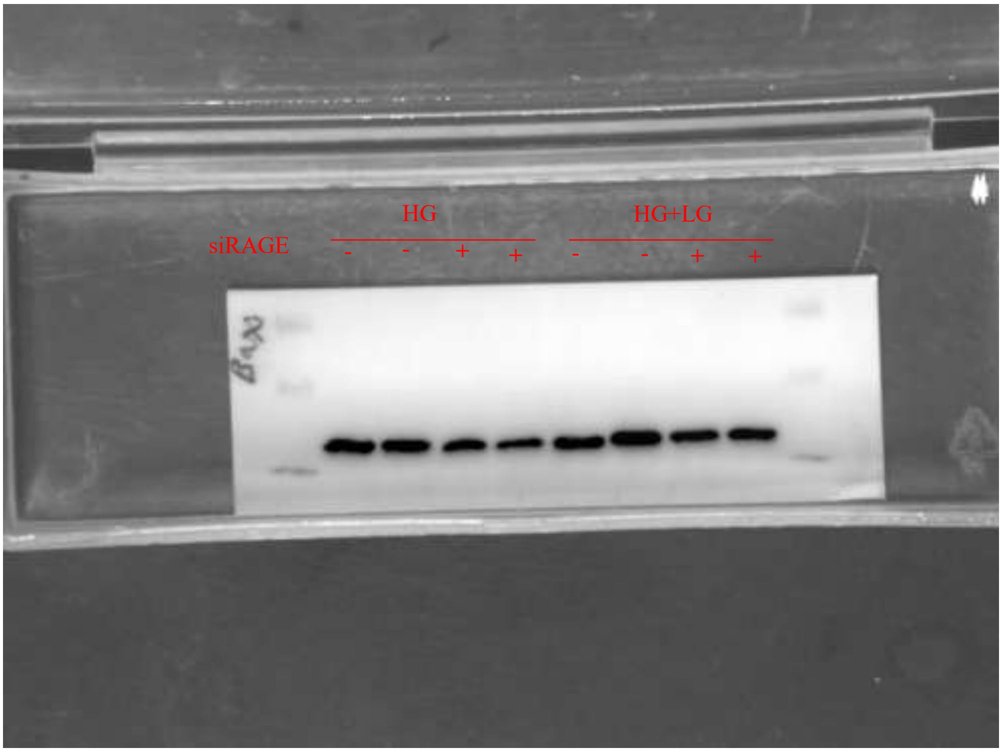

24kd

Figure5i

Caspase3

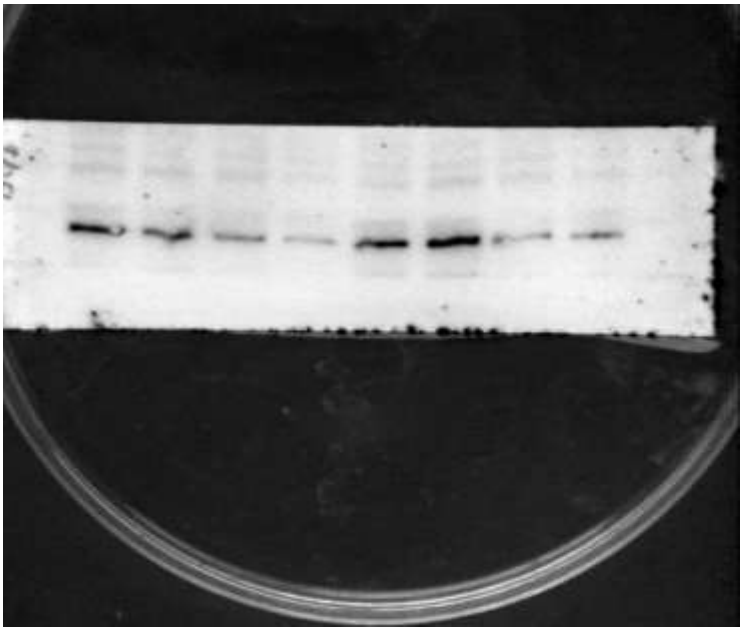

35kd

Figure5i

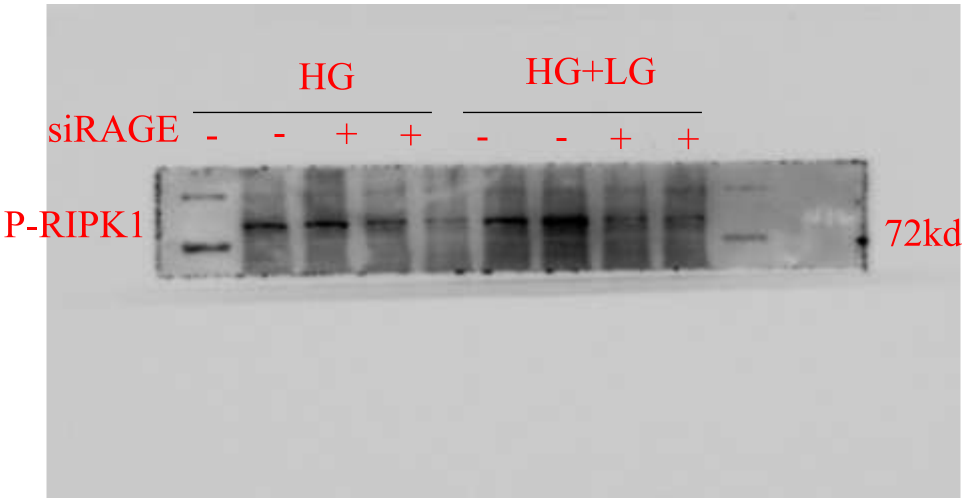

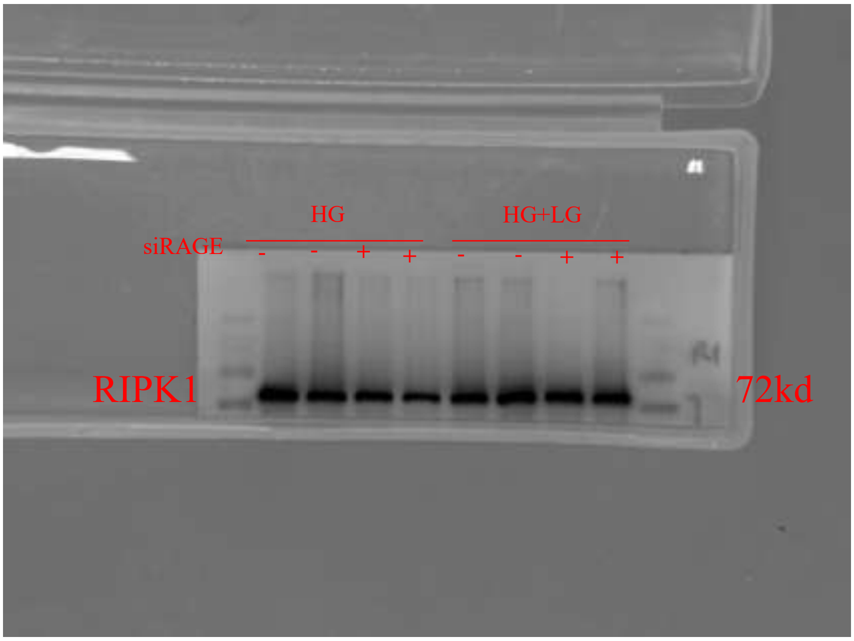

Figure5i

P-RIPK3

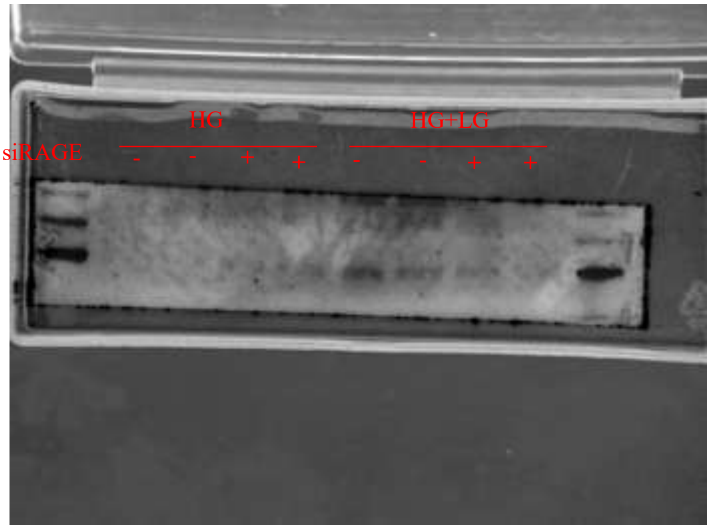

57kd

RIPK3

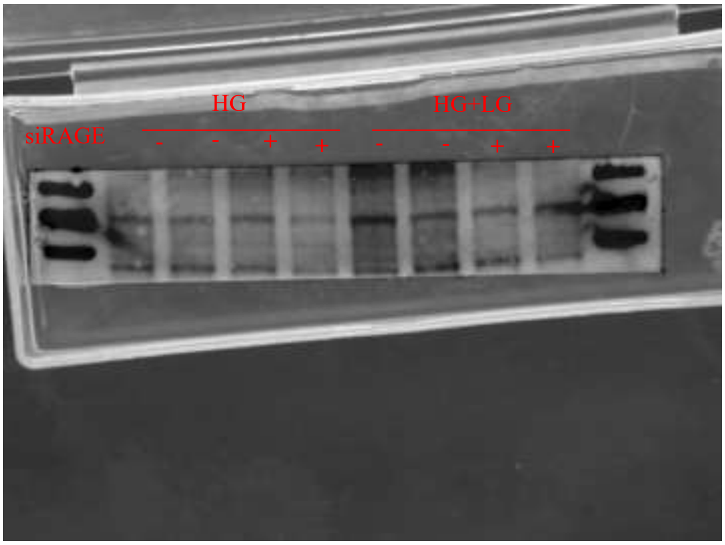

57kd

β-actin

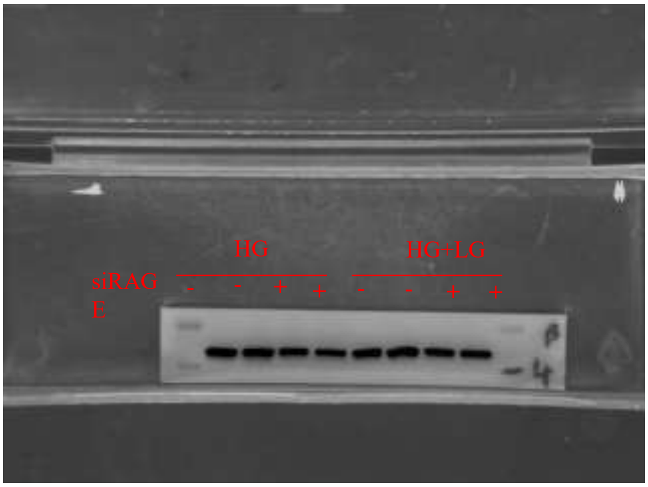

42kd

AGER NC-siRAGE 3.6S merge

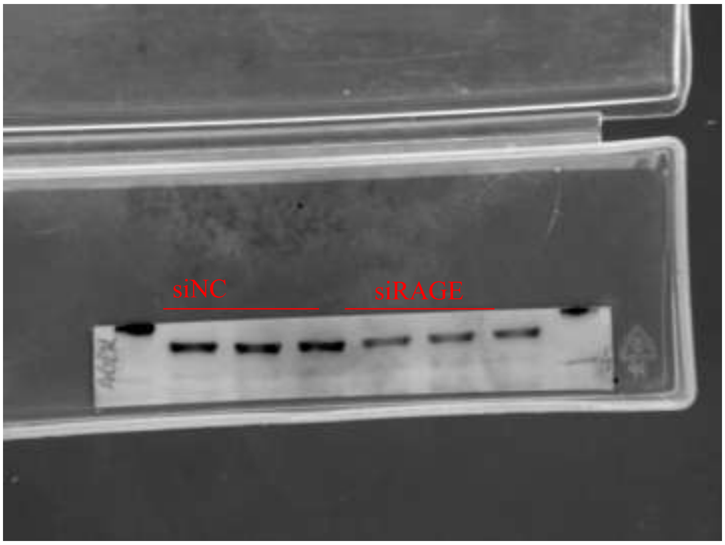

B-ACTIN NC-siAGER 0.1S merge

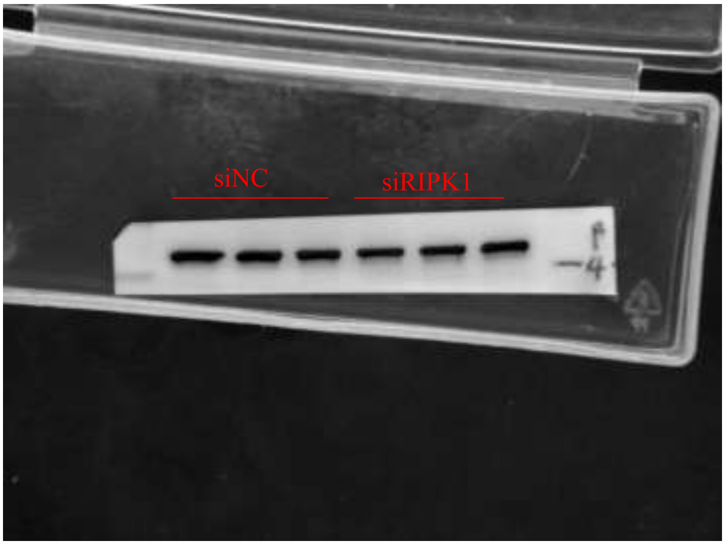

Supplementary Figure

RIPK1 NC-siRIPK1 3S MERGE

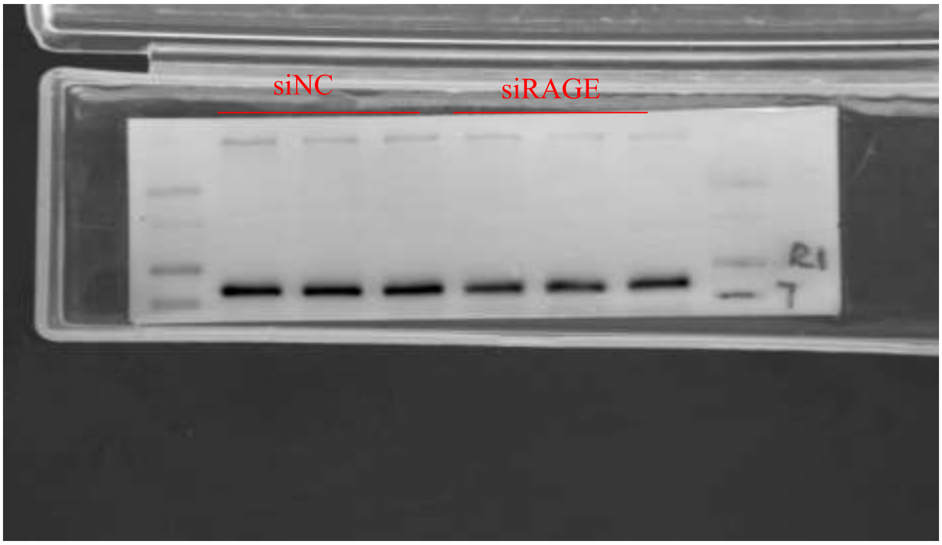

β-actin NC-siRIPK1 3S MERGE

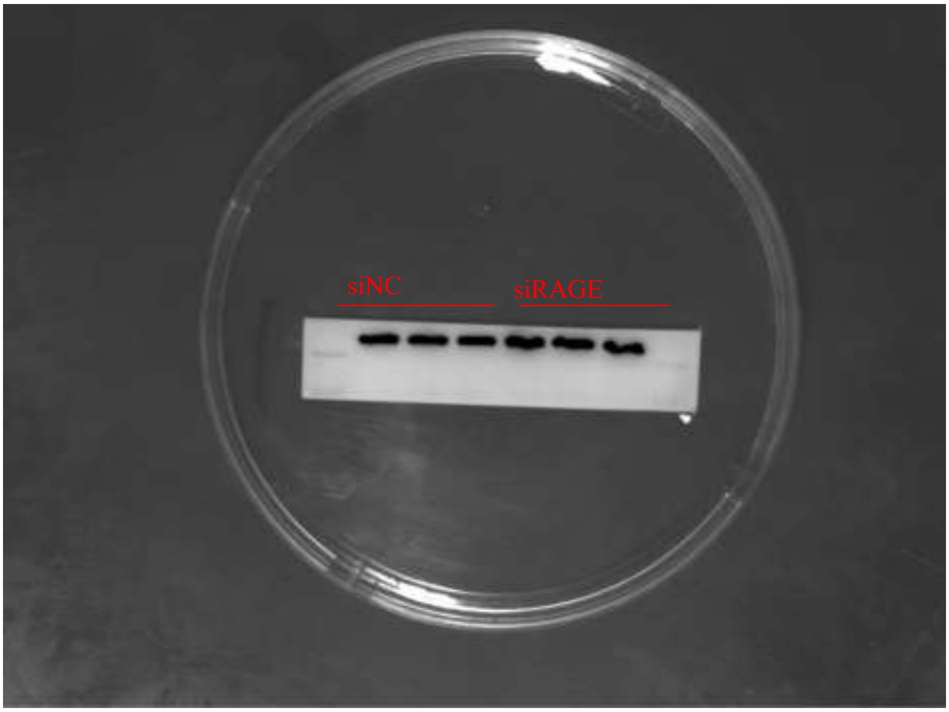

AIM2 NC-siAIM2 3S MERGE

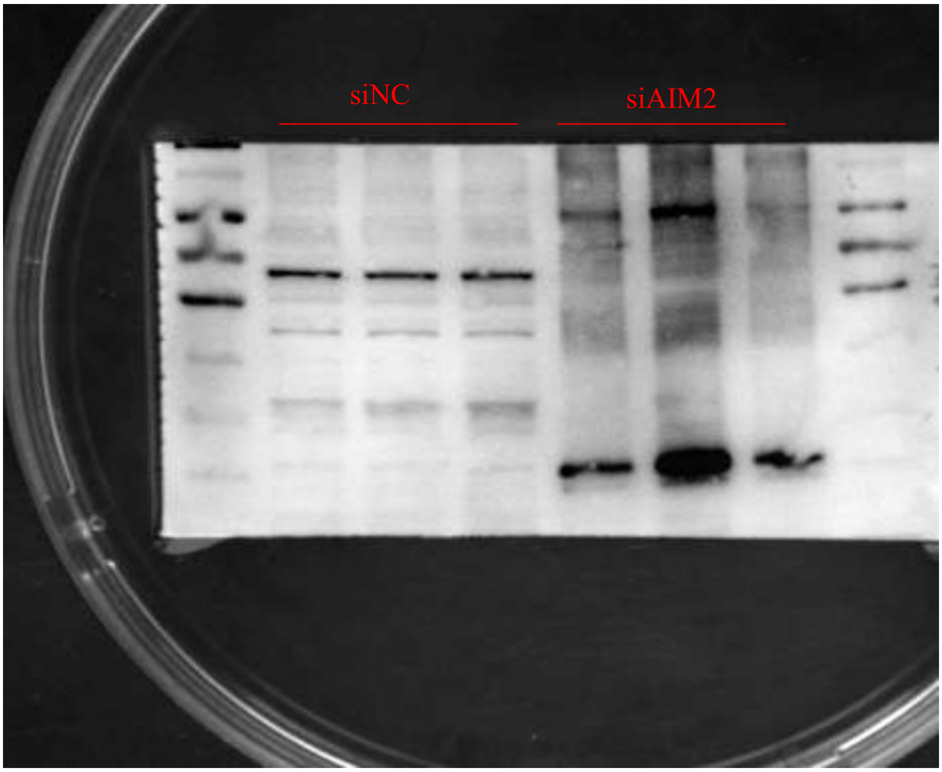

b-actin NC-siAIM2 0.2S MERGE

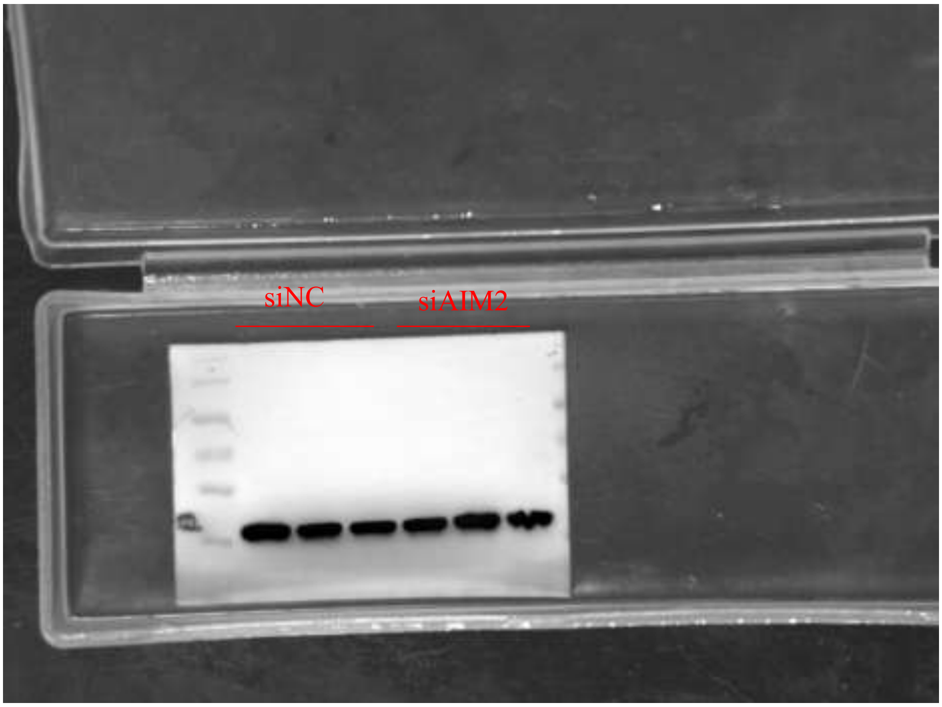

Supplement: Supplementary file 1 [file DataSheet1.pdf]
